# MicroRNA-mediated epigenetic targeting of Survivin significantly enhances the antitumor activity of paclitaxel against non-small cell lung cancer

**DOI:** 10.18632/oncotarget.9264

**Published:** 2016-05-10

**Authors:** Shuiliang Wang, Ling Zhu, Weimin Zuo, Zhiyong Zeng, Lianghu Huang, Fengjin Lin, Rong Lin, Jin Wang, Jun Lu, Qinghua Wang, Lingjing Lin, Huiyue Dong, Weizhen Wu, Kai Zheng, Jinquan Cai, Shunliang Yang, Yujie Ma, Shixin Ye, Wei Liu, Yinghao Yu, Jianming Tan, Bolin Liu

**Affiliations:** ^1^ Fujian Key Laboratory of Transplant Biology, Fuzhou General Hospital, Xiamen University, Fuzhou, Fujian, China; ^2^ Department of Thoracic Surgery, Fuzhou General Hospital, Xiamen University, Fuzhou, Fujian, China; ^3^ Department of Pathology, Fuzhou General Hospital, Xiamen University, Fuzhou, Fujian, China; ^4^ Department of Pathology, School of Medicine, University of Colorado Anschutz Medical Campus, Aurora, CO, USA

**Keywords:** entinostat, Survivin, miRNA, DNMT1, paclitaxel

## Abstract

Elevated expression of Survivin correlates with poor prognosis, tumor recurrence, and drug resistance in various human cancers, including non-small cell lung cancer (NSCLC). The underlying mechanism of Survivin upregulation in cancer cells remains elusive. To date, no Survivin-targeted therapy has been approved for cancer treatment. Here, we explored the molecular basis resulting in Survivin overexpression in NSCLC and investigated the antitumor activity of the class I HDAC inhibitor entinostat in combination with paclitaxel. Our data showed that entinostat significantly enhanced paclitaxel-mediated anti-proliferative/anti-survival effects on NSCLC cells *in vitro* and *in vivo*. Mechanistically, entinostat selectively decreased expression of Survivin via induction of miR-203 (*in vitro* and *in vivo*) and miR-542-3p (*in vitro*). Moreover, analysis of NSCLC patient samples revealed that the expression levels of miR-203 were downregulated due to promoter hypermethylation in 45% of NSCLC tumors. In contrast, increased expression of both DNA methytransferase I (DNMT1) and Survivin was observed and significantly correlated with the reduced miR-203 in NSCLC. Collectively, these data shed new lights on the molecular mechanism of Survivin upregulation in NSCLC. Our findings also support that the combinatorial treatments of entinostat and paclitaxel will likely exhibit survival benefit in the NSCLC patients with overexpression of DNMT1 and/or Survivin. The DNMT1-miR-203-Survivin signaling axis may provide a new avenue for the development of novel epigenetic approaches to enhance the chemotherapeutic efficacy against NSCLC.

## INTRODUCTION

Lung cancer remains the leading cause of cancer-related deaths worldwide [1, 2]. The current treatment for lung cancer patients who have been diagnosed at an early stage is surgical resection followed by chemotherapy. However, majority of the patients will eventually experience disease progression and require further treatment [3]. Although a number of new therapies have been developed for the patients with non-small cell lung cancer (NSCLC) [4], which accounts for 85% to 90% of all lung cancer cases [5], the 5-year survival rate of patients with advanced NSCLC remains low [6].

Survivin, the smallest member of IAP (inhibitor of apoptosis) family, is overexpressed in tumors, but not normal tissues [7, 8]. Increased Survivin correlates with poor prognosis, tumor recurrence, and drug resistance in a wide variety of human cancers, including NSCLC [9, 10]. Several approaches, such as the transcriptional inhibitor of *Survivin* YM155, antisense oligonucleotides, immunotherapy, and gene therapy have been designed to inhibit Survivin for cancer treatment [10, 11]. YM155 is the most advanced studied and actively under clinical evaluations (http://www.clinicaltrial.gov/ct2/results?term=YM155&Search=Search). A recent Phase II trial indicates that YM155 is safe, but it fails to increase the response rates to chemotherapy in patients with advanced NSCLC [12]. This failure is likely due to YM155′s insufficient inhibition of Survivin. It is believed that novel strategy effectively downregulating Survivin is required to enhance chemotherapeutic efficacy in the treatment of NSCLC patients. Nonetheless, the underlying mechanism of Survivin upregulation in NSCLC remains unclear.

Currently, the platinum-based doublet regimens containing paclitaxel and cisplatin are the standard of care for advanced NSCLC [13]. Paclitaxel, either as monotherapy or combined with other agents, has shown potent anti-NSCLC activity [14, 15]. However, both *de novo* and acquired resistance to paclitaxel frequently occurs and represents a huge clinical problem [16]. We showed that overexpression of erbB3 upregulated Survivin to confer paclitaxel resistance in erbB2-overexpressing breast cancer cells [17]. Inhibition of Survivin via a shRNA or an anti-erbB3 antibody (Ab) significantly increased the cytotoxicity of paclitaxel [17, 18]. In identifying novel approach targeting of erbB3, we discovered that the class I histone deacetylase (HDAC) inhibitor entinostat specifically increased miR-125a, miR-125b, and miR-205, which acted in concert to inhibit erbB3, and subsequently induced apoptosis in erbB2-overexpressing breast cancer cells [19, 20]. Entinostat exerts potent antitumor activity in a number of cancers [21]. Its clinic activity against NSCLC is being tested in combination with DNA methyltransferase (DNMT) inhibitor Azacitidine or EGFR inhibitor Erlotinib (Tarceva), but not conventional chemotherapy (http://www.clinicaltrial.gov/ct2/results?term=entinostat&Search=Search). While epigenetic therapy emerges as a new strategy to overcome drug resistance and re-sensitize cancer cells to chemotherapy [22, 23], we wondered whether entinostat might possess such activity as a chemo-sensitizer via inhibition of Survivin. In the current report, we investigated the mechanism of action of entinostat in potentiation of paclitaxel-mediated antitumor activity against NSCLC. We have also explored the molecular basis responsible for Survivin overexpression in NSCLC.

## RESULTS

### Entinostat enhances the anti-proliferative/anti-survival effects of paclitaxel on NSCLC cells

**Figure 1:**
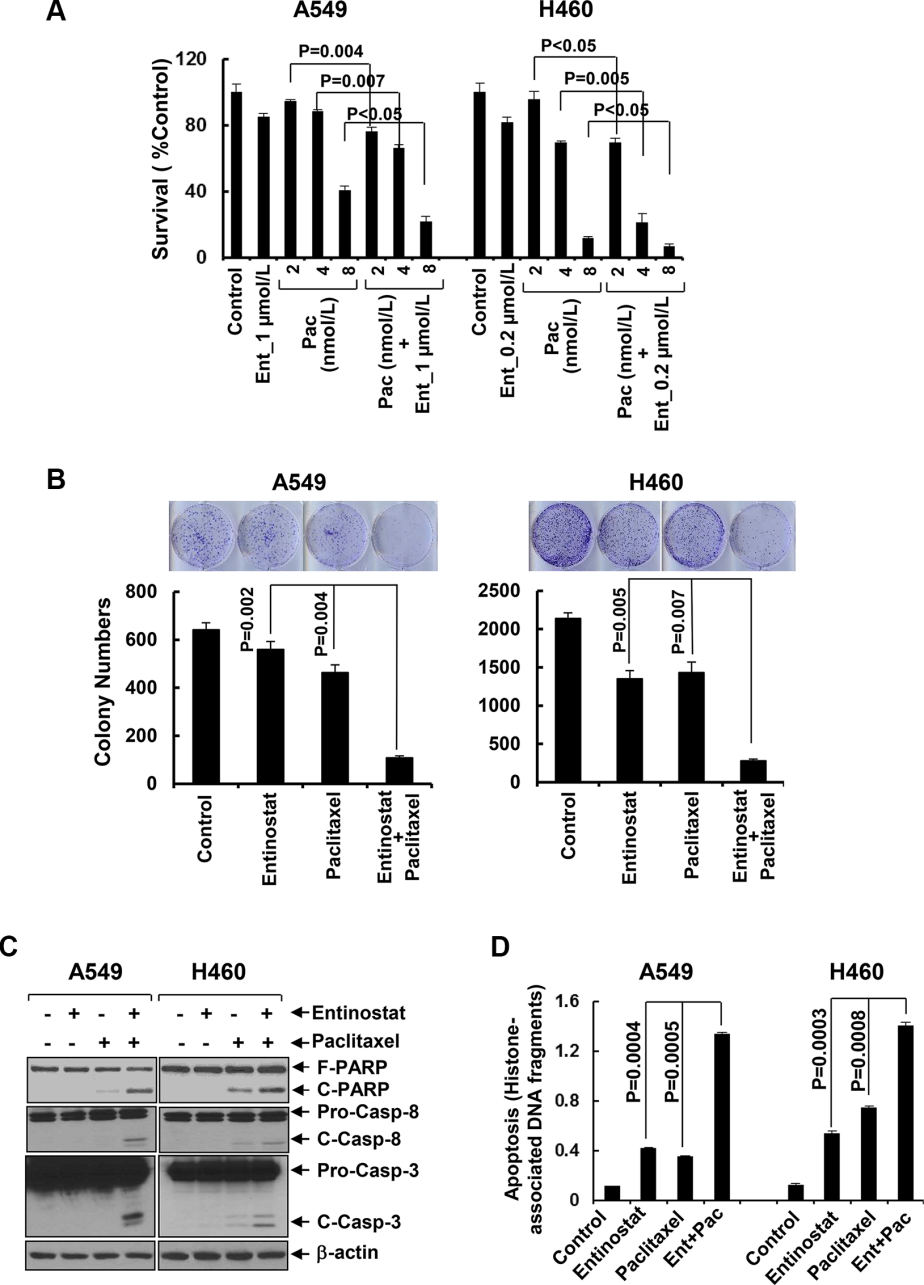
Entinostat significantly enhances paclitaxel-induced growth inhibition and apoptosis in NSCLC cells (**A**) A549 and H460 cells were plated onto 96-well plates. After 24 h, the culture media were replaced with fresh media or the same media containing indicated concentrations of either entinostat (Ent) or paclitaxel (Pac) or both entinostat and paclitaxel for another 72 h. The percentages of surviving cells from each cell line relative to controls, defined as 100%, were determined by reduction of MTS. (**B**) A549 and H460 cells were grown in triplicates in the absence or presence of either entinostat (1 μmol/L for A549 and 0.2 μmol/L for H460) or paclitaxel (4 nmol/L) alone or both entinostat and paclitaxel for 2–3 weeks. The pictures and numbers of the cell colonies were obtained by the QuantiOne software of Fluor-S™ Multimager. (**C** and **D**) A549 and H460 cells were treated with either entinostat (3 μmol/L and 0.5 μmol/L for A549 and H460, respectively), or paclitaxel (6 nmol/L and 3 nmol/L for A549 and H460, respectively) alone, or both entinostat and paclitaxel for 24 h. Cells were collected and subjected to western blot analyses of PARP (F-PARP, full length PARP; C-PARP, cleaved PARP), caspase-8 (Pro-Casp-8, pro-caspase-8; C-Casp-8, cleaved caspase-8), caspase-3 (Pro-Casp-3, pro-caspase-3; C-Casp-3, cleaved caspase-3), or β-actin (C), or apoptotic-ELISA (D). *Bars*, S.D. All data show the representative of three independent experiments.

To explore the therapeutic potential of entinostat against NSCLC, we first studied the inhibitory activity of entinostat combined with paclitaxel in NSCLC cell lines. While entinostat alone slightly reduced proliferation of A549 and H460 cells, it significantly increased paclitaxel-mediated growth inhibition in both lines (Figure 1A). This data was supported by clonogenic assays showing that combinations of entinostat and paclitaxel as compared to either agent alone dramatically reduced the colony numbers (Figure 1B). To evaluate whether the combinatorial inhibition via a similar mechanism observed in breast cancer and multiple myeloma cells [24, 25], we examined the effects of entinostat and/or paclitaxel on induction of apoptosis. Treatment of A549 or H460 cells with both entinostat and paclitaxel, but not either agent alone, clearly induced PARP cleavage, a hallmark of apoptosis, and activation of caspase-8 and caspase-3 (Figure 1C). An ELISA measuring histone-associated DNA fragments further confirmed that the cells underwent apoptotic cell death upon the combinatorial treatments (Figure 1D). These data indicate that entinostat significantly enhances paclitaxel-induced anti-proliferative/anti-survival effects on NSCLC cells associated with caspase-dependent apoptosis.

### Entinostat selectively inhibits Survivin independent of PI-3K/Akt/mTOR signaling

**Figure 2:**
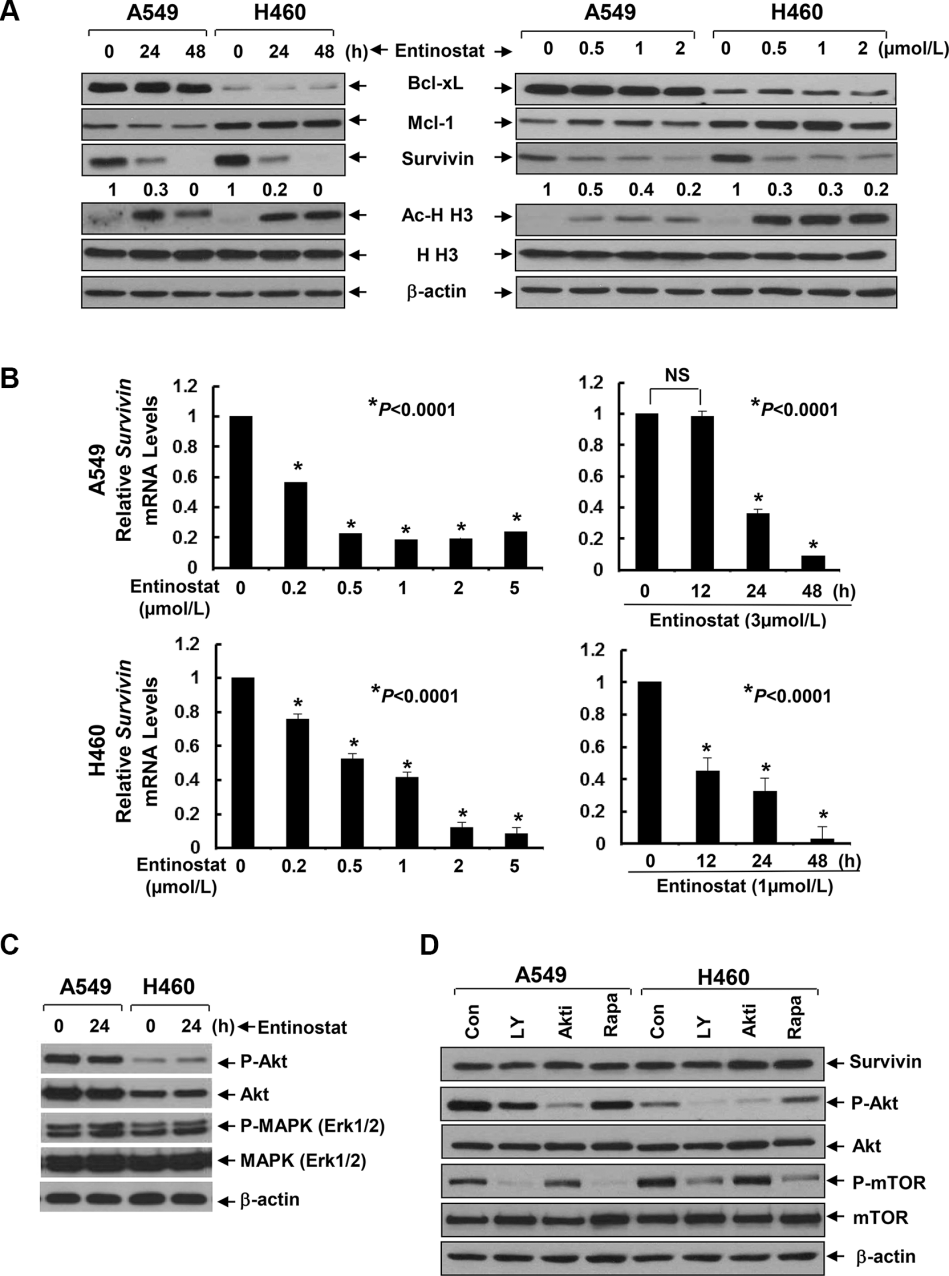
Entinostat reduces both the protein and mRNA levels of *Survivin* in NSCLC cells without disturbing the PI-3K/Akt and MEK/MAPK signaling pathways (**A**) A549 and H460 cells were treated with either entinostat (3 μmol/L and 0.5 μmol/L for A549 and H460, respectively) for 24 or 48 h (left panel), or indicated concentrations of entinostat for 24 h (right panel). Cells were collected and subjected to western blot analyses of Bcl-xL, Mcl-1, Survivin, Acetyl-Histone H3 (Ac-H H3), Histone H3 (H H3), or β-actin. The densitometry analyses of Survivin signals were shown underneath, and the arbitrary numbers indicate the intensities of each cell line relative to controls, defined as 1.0. (**B**) A549 and H460 cells were treated with either indicated concentrations of entinostat for 24 h (left panel), or a fixed concentration of entinostat for 12, 24, or 48 h (right panel). Cells were collected and subjected to total RNA extraction. The mRNA levels of *Survivin* were measured by qRT-PCR. All results were normalized with the internal control β-actin. *Bars*, S.D. Data show the representative of three independent experiments. (**C**) Cells treated with entinostat (3 mmol/L and 0.5 mmol/L for A549 and H460, respectively) for 24 h were collected and subjected to western blot analyses of P-Akt, Akt, P-MAPK (Erk1/2), MAPK (Erk1/2), or β-actin. (**D**) A549 and H460 cells treated with LY294002 (LY, 10 μmol/L), Akt inhibitor VIII (Akti, 1 μmol/L), or Rapamycin (Rapa, 100 nmol/L) for 24 h were subjected to western blot analyses of Survivin, P-Akt, Akt, P-mTOR, mTOR, or β-actin.

**Figure 3:**
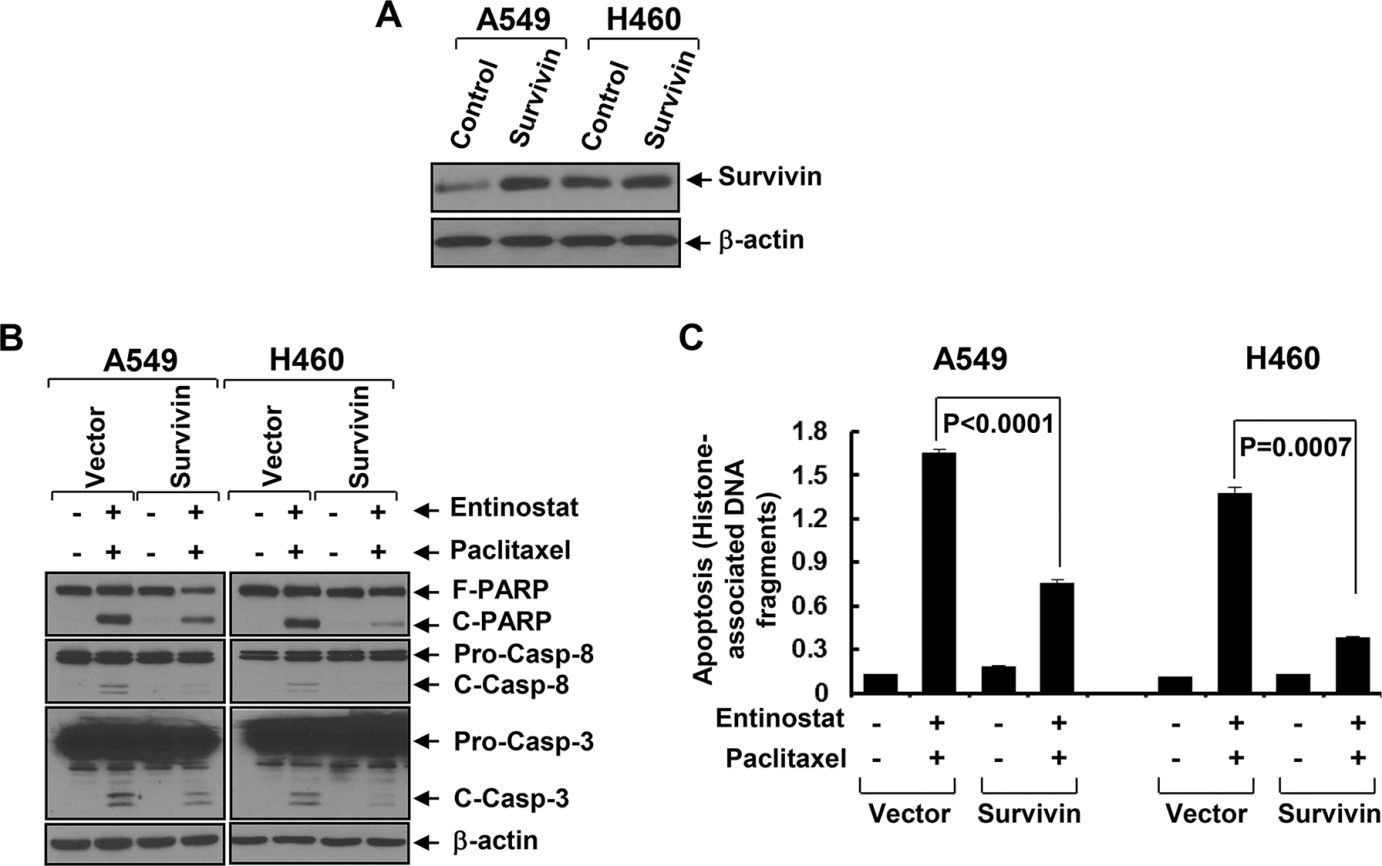
Ectopic expression of Survivin significantly attenuates entinostat potentiation of paclitaxel-induced apoptosis A549 and H460 cells infected with lentivirus containing either control vector pLEX-MCS (Control) or pLEX-*hSurvivin* (Survivin) were subjected to the following experiments. (**A**) Western blot analyses of Survivin or β-actin. (**B** and **C**) Cells were then treated with combinations of entinostat (3 μmol/L and 0.5 μmol/L for A549 and H460, respectively) and paclitaxel (6 nmol/L and 3 nmol/L for A549 and H460, respectively) for 24 h. Cells were collected and subjected to western blot analyses of PARP (F-PARP, full length PARP; C-PARP, cleaved PARP), caspase-8 (Pro-Casp-8, pro-caspase-8; C-Casp-8, cleaved caspase-8), caspase-3 (Pro-Casp-3, pro-caspase-3; C-Casp-3, cleaved caspase-3), or β-actin (B), or apoptotic-ELISA (C). *Bars*, S.D.

To explore the mechanism by which entinostat potentiates paclitaxel-induced apoptosis in NSCLC cells, we first examined several anti-apoptosis proteins, including Bcl-xL, Mcl-1, and Survivin in A549 and H460 cells. Entinostat markedly decreased both protein and mRNA levels of Survivin, but not Bcl-xL and Mcl-1, in a time- and dose-dependent manner (Figure 2A and 2B). Although entinostat inhibited PI-3K/Akt signaling and activation of the PI-3K/Akt pathway led to Survivin upregulation in breast cancer cells [17, 19], it had no effect on phosphorylation of Akt (P-Akt) and MAPK (P-MAPK) in NSCLC cells (Figure 2C). Consistently, specific inhibitor of PI-3K (LY294002), Akt (Akt1/2 inhibitor VIII), or mTOR (rapamycin) did not alter Survivin expression (Figure 2D). We next studied whether the reduction of Survivin played a causal role for entinostat to potentiate paclitaxel-induced apoptosis. Ectopic expression of Survivin in A549 or H460 cells abrogated entinostat enhancement of paclitaxel-induced PARP cleavage, activation of caspase-8 and −3 and DNA fragmentation (Figure 3). Finally, we tested whether Survivin reduction was able to enhance the cytotoxicity of paclitaxel. Two verified shRNA sequences were used to downregulate Survivin [17]. As expected, specific knockdown of Survivin not only enhanced paclitaxel-mediated growth inhibition, but also significantly increased paclitaxel-induced apoptosis (Supplementary Figure S1). Collectively, our data demonstrate that entinostat elicits a PI-3K/Akt/mTOR pathway-independent mechanism to downregulate Survivin, which is essential for entinostat to potentiate paclitaxel-mediated cytotoxicity in NSCLC cells.

### Entinostat downregulates Survivin via induction of miR-203 and miR-542-3p

**Figure 4:**
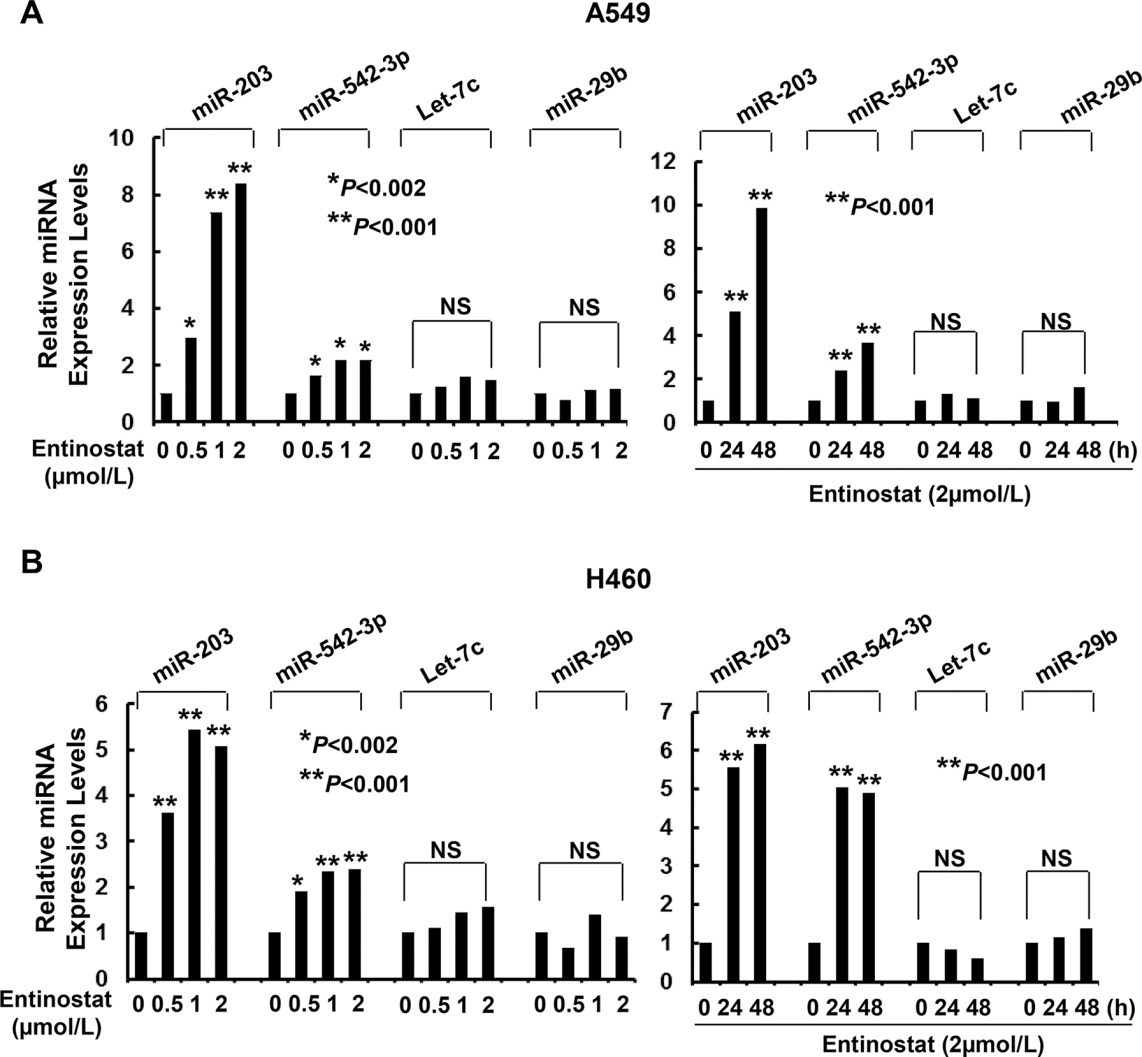
Entinostat selectively increases the expression levels of miR-203 and miR-542-3p in A549 and H460 cells (**A**) A549 and (**B**) H460 cells untreated or treated with entinostat (0.5, 1, or 2 μmol/L) for 24 hr, or with entinostat (2 μmol/L) for 24 or 48 h were collected and subjected to total RNA extraction, inclusive of the small RNA fraction. The expression levels of miR-203, miR-542-3p, Let-7c, and miR-29b were measured by qRT-PCR using TaqMan miRNA assays. All results were normalized with the internal control RNU6B. *Bars*, S.D. Data show the representative of three independent experiments. NS, no significance

**Figure 5:**
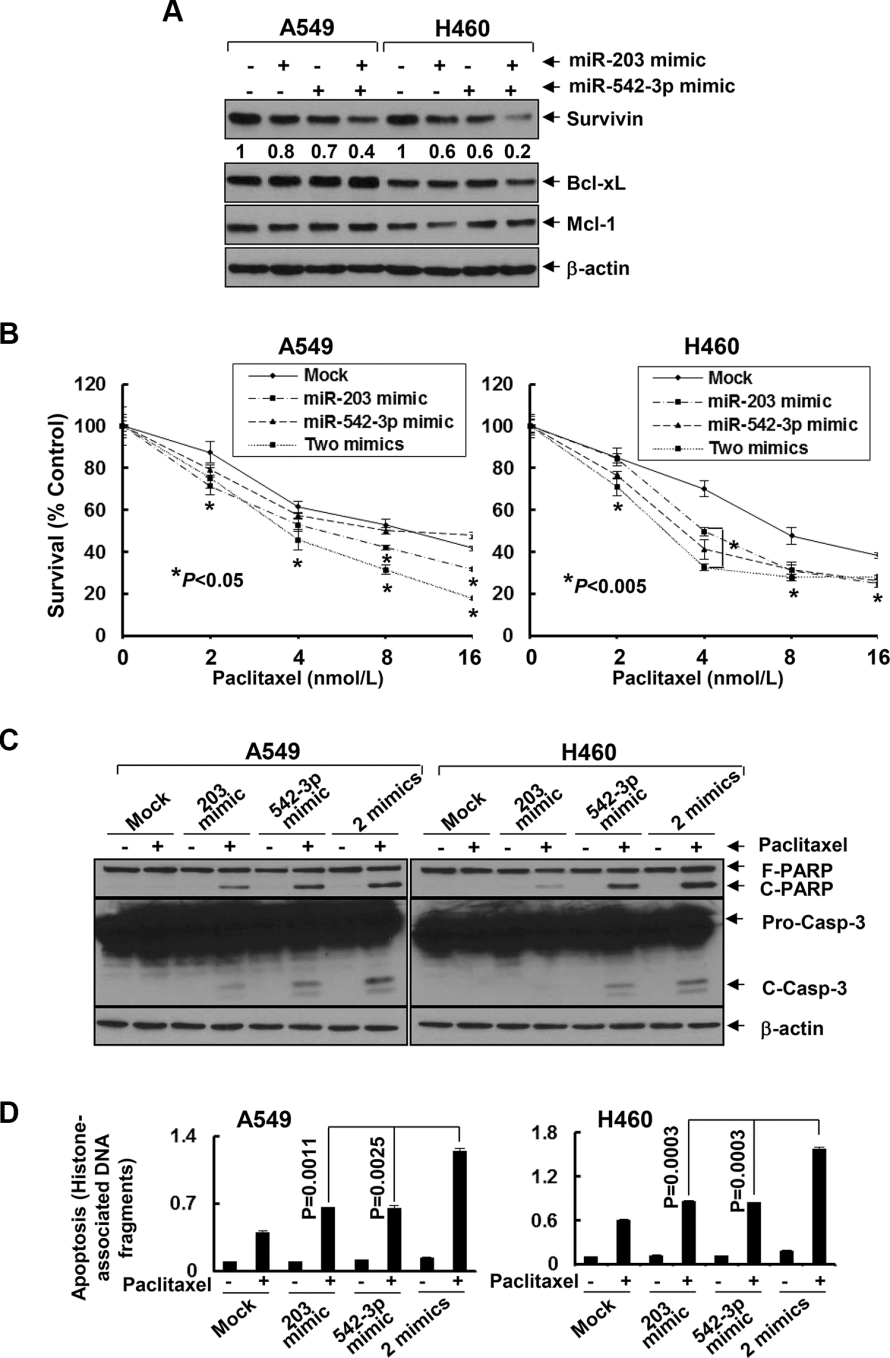
MiR-203 and/or miR-542-3p mimics specifically downregulate Survivin and significantly enhance paclitaxel-induced growth inhibition and apoptosis in NSCLC cells A549 and H460 cells were either mock transfected or transfected with miR-203 mimic (40 nmol/L), or miR-542-3p mimic (10 nmol/L), or combinations of two miRNA mimics. (**A**) After 24 h, some cells were harvested and subjected to western blot analyses of Survivin, Bcl-xL, Mcl-1, or β-actin. The densitometry analyses of Survivin signals were shown underneath, and the arbitrary numbers indicate the intensities of each cell line relative to controls, defined as 1.0. (**B**) Some transfected cells were then plated onto 96-well plates and incubated for 24 h. The culture media were then replaced with either fresh media or the same media containing indicated concentrations of paclitaxel for additional 72 h. The percentages of surviving cells from each cell line relative to controls, defined as 100% survival, were determined by reduction of MTS. (**C** and **D**) Some transfected cells were then untreated or treated with paclitaxel (6 nmol/L and 3 nmol/L for A549 and H460, respectively) for 24 h. Cells were collected and subjected to western blot analyses of PARP, caspase-3, or β-actin (C), or apoptotic-ELISA (D). *Bars*, S.D. Data show the representative of three independent experiments.

**Figure 6:**
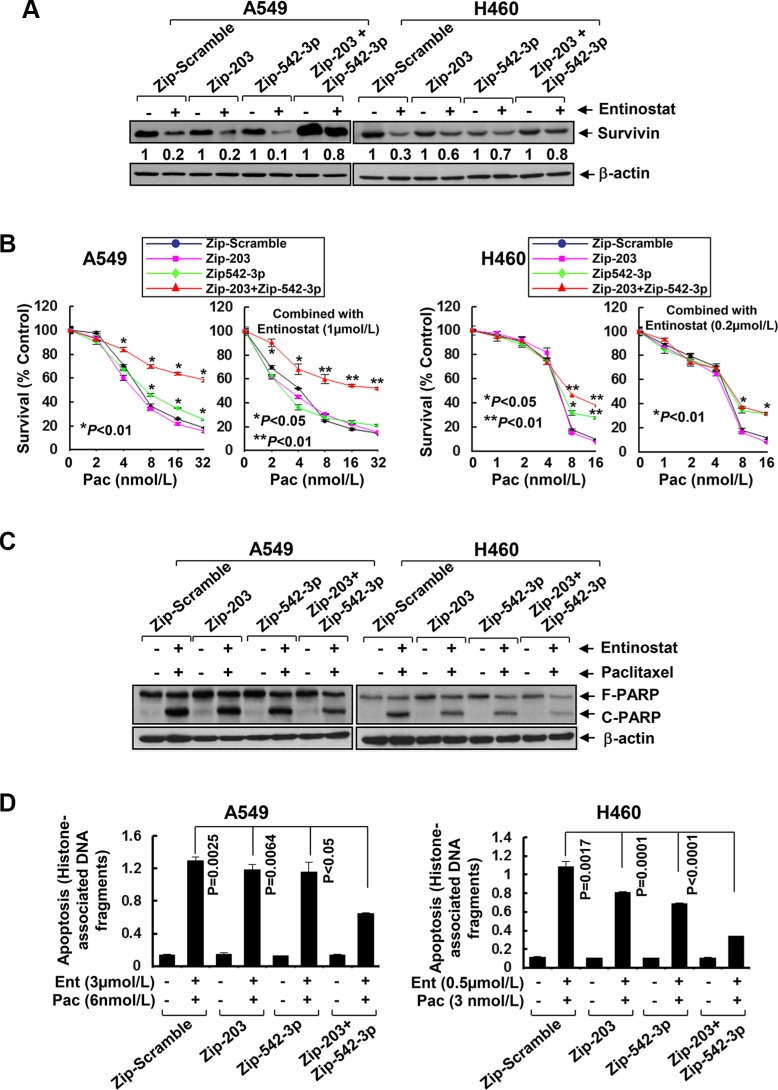
Specific knockdown of miR-203 and/or miR-542-3p reverses entinostat-enhanced paclitaxel-mediated growth inhibition and apoptosis in NSCLC cells A549 and H460 cells were infected with single lentiviral miRZip™ expression system of either scramble control, or miRZip-203, or miRZip-542-3p, or co-infected with both miRZip-203 and miRZip-542-3p. All infected cells were subjected to selection with puromycin (1 μg/mL) for two weeks. (**A**) Cells with stable knockdown of miR-203 and/or miR-542-3p were then treated with entinostat (0.5 μmol/L for both A549 and H460) for 24 h. Cells were collected and subjected to western blot analyses of Survivin or β-actin. The densitometry analyses of Survivin signals were shown underneath, and the arbitrary numbers indicate the intensities of each cell line relative to controls, defined as 1.0. (**B**) Cells with stable knockdown of miR-203 and/or miR-542-3p were plated onto 96-well plates. After 24 h, the culture media were replaced with fresh media or the same media containing either indicated concentrations of paclitaxel alone or in combination with entinostat for another 72 h. The percentages of surviving cells from each cell line relative to controls, defined as 100% survival, were determined by reduction of MTS. (**C** and **D**) Cells with stable knockdown of miR-203 and/or miR-542-3p were treated with the combinations of entinostat (3 μmol/L and 0.5 μmol/L for A549 and H460, respectively) and paclitaxel (6 nmol/L and 3 nmol/L for A549 and H460, respectively) for 24 h. Cells were collected and subjected to western blot analyses of PARP or β-actin (C), or apoptotic-ELISA (D). *Bars*, S.D. Data show the representative of three independent experiments.

As miRNAs emerge as important regulators of gene expression by targeting mRNA for degradation or translational repression [26], we asked if entinostat might modulate specific miRNAs to control Survivin expression in NSCLC cells. Quantitative real-time (qRT)-PCR showed that entinostat significantly induced miR-203 and miR-542-3p, two *Survivin*-targeting miRNAs [27, 28], in a dose- and time-dependent manner (Figure 4). It had no effect on Let-7c and miR-29b, which have been reported to target *Bcl-xL* and *Mcl-1*, respectively [29, 30]. It appeared that chemotherapy alone had no effect on the miRNAs *in vitro*, as treatment of A549 or H460 cells with paclitaxel did not alter the expression of either miR-203 or miR-542-3p (Data not shown). We next explored whether the induction of miR-203 and/or miR-542-3p was sufficient to inhibit Survivin. While transfection with the mimic of either miR-203 or miR-542-3p slightly decreased Survivin, the combinations of both miRNA mimics profoundly reduced Survivin in A549 and H460 cells (Figure 5A). It seemed that miR-542-3p or two miRNA mimic(s) were more effective than miR-203 mimic to enhance paclitaxel-induced growth inhibition (Figure 5B). Moreover, two mimics exhibited a more potent activity than single mimic to significantly potentiate paclitaxel-induced apoptosis (Figure 5C and 5D). The miRNA mimics largely recapitulated entinostat's activity to enhance paclitaxel cytotoxicity. We then sought to understand if the induction of miR-203 and/or miR-542-3p was required for entinostat-induced downregulation of Survivin. The lentiviral miRZip-203 or miRZip-542-3p was applied to specifically inhibit miR-203 or miR-542-3p, respectively. High efficiency of lentiviral infection of NSCLC cells was evidenced by visualization of GFP (Supplementary Figure S2). While either miRZip-203 or miRZip-542-3p had little effect on entinostat-mediated reduction of Survivin, inhibition of both miR-203 and miR-542-3p attenuated entinostat's inhibitory effect on Survivin (Figure 6A), suggesting that miR-203 and miR-542-3p worked cooperatively in entinostat action to downregulate Survivin. As a consequence, simultaneous inhibition of miR-203 and miR-542-3p significantly reversed entinostat enhancement of paclitaxel-induced growth inhibition and apoptosis (Figure 6B–6D). Taken together, our data indicate that both miR-203 and miR-542-3p contribute to entinostat-mediated reduction of Survivin; and upregulation of the two miRNAs is necessary and sufficient for entinostat potentiation of paclitaxel-induced growth inhibition and apoptosis in NSCLC cells.

### Entinostat enhances the antitumor activity of paclitaxel against NSCLC *in vivo*

**Figure 7:**
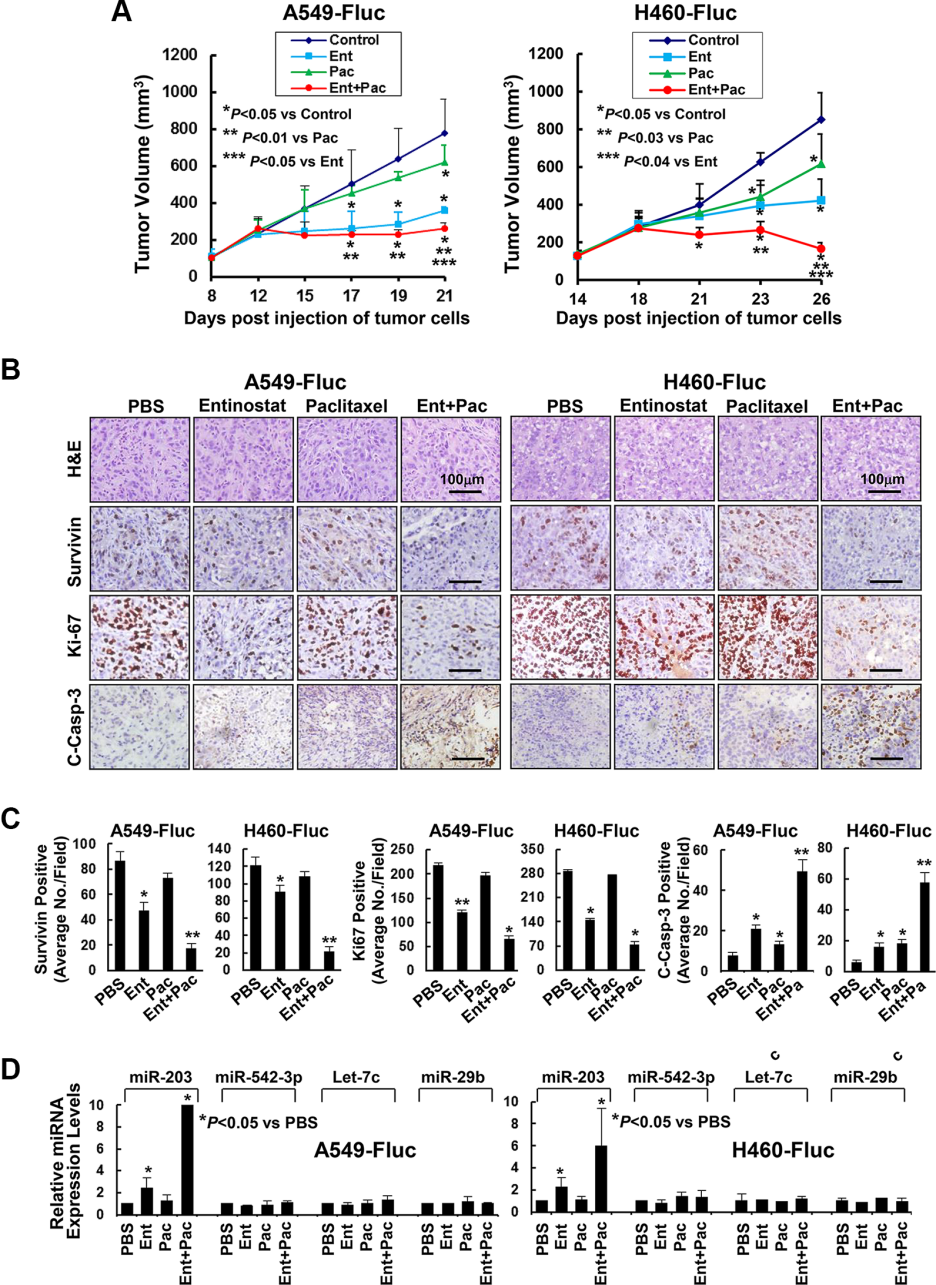
Entinostat in combination with paclitaxel significantly inhibits *in vivo* tumor growth of NSCLC cells A549-Fluc or H460-Fluc cells were subcutaneously inoculated into nude mice to establish tumor xenografts. The tumor-bearing mice received i.p. injections of either PBS, or entinostat (25 mg/kg for A549-Fluc, 12.5 mg/kg for H460-Fluc), or paclitaxel (7.5 mg/kg) alone, or both entinostat and paclitaxel as described in Methods. After five treatments, all mice were euthanized and their tumors were excised for histology, IHC, and miRNAs expression analyses. (**A**) The graphs show the tumor growth curves. *Bars*, S.D. (**B**) Data show the representative tumors with H&E staining and IHC analysis of Survivin, Ki-67, and cleaved caspase-3 (C-Casp-3). (**C**) The IHC slides were observed by two independent personnel. The tumor cells with positive staining of Survivin, Ki-67, or cleaved caspase-3 were counted from three randomly selected areas in each slide. The three areas were first identified by scanning the entire slide at × 100 magnification, and then the positive-stained cells were counted at × 200 magnification using an Olympus BX40 microscope. The bar graphs show the average of positive staining cells in each field. *Bars*, S.D. *P* values versus control were indicated. *P < 0.01, **P < 0.001. (**D**) Isolated tumors were immersed in *RNA* safer reagent and subjected to total RNA extraction, inclusive of the small RNA fraction. The expression levels of miR-203, miR-542-3p, Let-7c, and miR-29b were measured by qRT-PCR. All results were normalized with the internal control RNU6B. *Bars*, S.D. Data show the representative of three independent experiments.

To determine whether entinostat holds potential to enhance paclitaxel's antitumor activity *in vivo*, we took advantage of tumor xenograft models established from A549 or H460 cells. The firefly luciferase-labelled A549 (A549-Fluc) and H460 (H460-Fluc) cells were first tested *in vitro*, and found that their luciferase activity was in a linear association with the cell numbers seeded (Supplementary Figure S3). We then injected A549-Fluc or H460-Fluc cells into nude mice to establish tumor xenografts. Tumor-bearing mice were then treated with intraperitoneal injection of either PBS, or entinostat, or paclitaxel, or both entinostat and paclitaxel. Paclitaxel slightly reduced tumor growth, whereas entinostat showed clear antitumor effects, consistent with previous findings [18, 21]. Importantly, the combinations of entinostat and paclitaxel exhibited a more potent antitumor activity than either agent (Figure 7A). The bioluminescence signals were monitored by IVIS-200 image system, which showed that treatment with both entinostat and paclitaxel profoundly reduced the luciferase activity (Supplementary Figure S4).

To examine the effects of entinostat and/or paclitaxel on proliferation and apoptosis *in vivo*, the tumors were subjected to IHC analyses of Ki-67, cleaved caspase-3, and Survivin. Mice treated with paclitaxel retained a similar number of tumor cells positively staining for Survivin or Ki-67 as control mice, whereas entinostat significantly reduced Survivin- and Ki-67-staining cells. Combinations of entinostat and paclitaxel further decreased Survivin- and Ki-67-positive cells. The tumors from mice treated with either entinostat or paclitaxel had a minor, but significant induction of cleaved caspase-3. However, entinostat combined with paclitaxel markedly increased cleaved caspase-3 (Figure 7B and 7C). Interestingly, entinostat significantly upregulated miR-203 (not miR-542-3p); and this induction was further enhanced when entinostat was combined with paclitaxel (Figure 7D). Thus, our studies indicate that entinostat, likely through upregulation of miR-203, potentiates the antitumor activity of paclitaxel against NSCLC via inhibition of cell proliferation and induction of apoptosis *in vivo*.

### Entinostat elicits hypomethylation of *miR-203* promoter and downregulation of DNMT1

**Figure 8:**
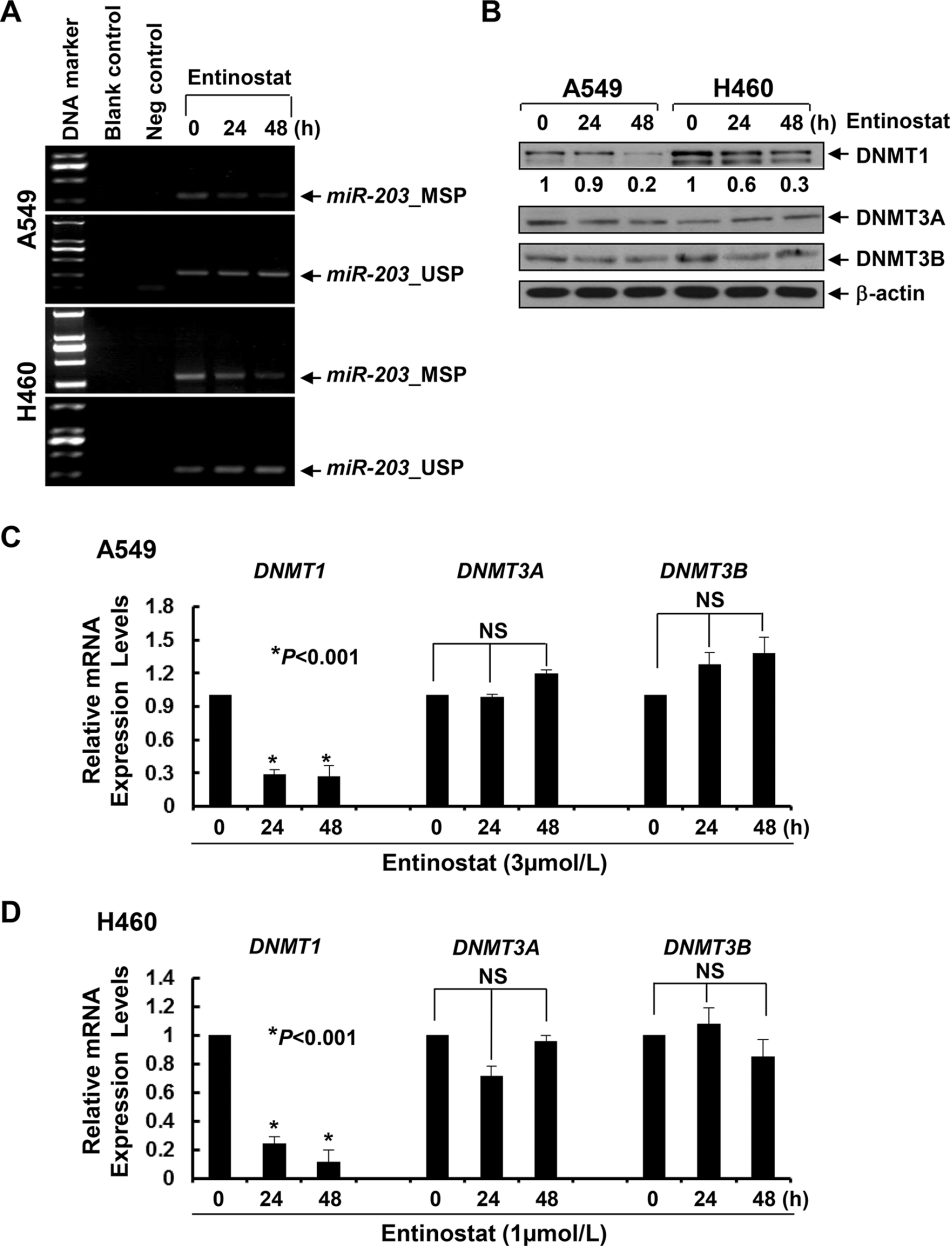
Entinostat demethylates *miR-203* promoter in NSCLC cells correlated with the downregulation of both protein and mRNA levels of DNMT1 *in vitro* A549 and H460 cells were untreated or treated with entinostat (3 μmol/L for A549, 1 μmol/L for H460) for 24 or 48 h. (**A**) Some cells were collected and subjected to DNA extraction. Methylation-specific PCR (MSP) and unmethylation-specific PCR (USP) analyses of *miR-203* promoter were performed as described in Methods. Blank control, double distilled H_2_O as PCR template. Negative control, unconverted genomic DNA of A549 or H460 cells as PCR template. (**B**) Some cells were subjected to western blot analyses of DNMT1, DNMT3A, DNMT3B, or β-actin. The densitometry analyses of DNMT1 signals were shown underneath, and the arbitrary numbers indicate the intensities of each cell line relative to controls, defined as 1.0. (**C** and **D**) One third of the cells were subjected to total RNA extraction. The mRNA levels of *DNMT1*, *DNMT3A*, and *DNMT3B* were measured by qRT-PCR. All results were normalized with the internal control *β-actin*. *Bars*, S.D. Data show the representative of three independent experiments. NS, no significance.

**Figure 9:**
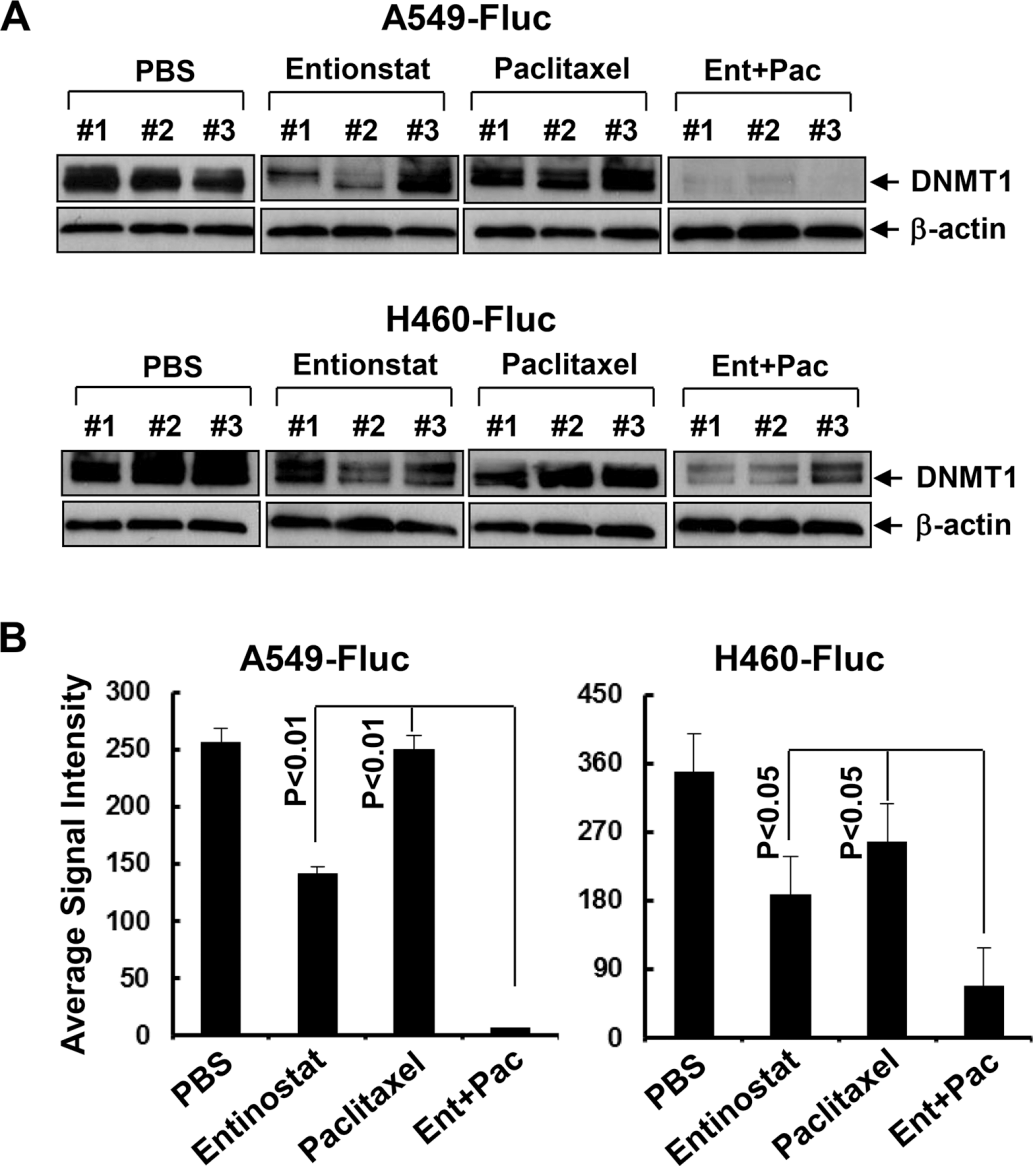
Entinostat reduces the expression of DNMT1 and its combination with paclitaxel exerts more potent inhibitory effects on DNMT1 *in vivo* (**A**) Tumor tissues obtained from the animal studies (Figure 7) were homogenized to generate protein lysates. Equal amount of the total lysates from three tumors of each treatment group were used for western blot analysis of DNMT1 or β-actin. (**B**) DNMT1 signal intensities of the tumors were obtained by densitometry analyses. The average signal intensity of each group was shown. *Bars*, S.D.

The expression of miR-203 is frequently downregulated due to promoter methylation in cancers of breast, prostate, liver, and hematologic malignancies [31–36]. To date, there is no such report in NSCLC. Although the expression levels of miR-203 are much lower in lung cancer cell lines than that in normal bronchial epithelial cells [37], it is unclear if this reduction is due to *miR-203* promoter methylation. Methylation-specific PCR (MSP) showed that treatment with entinostat lowered *miR-203* promoter methylation and increased unmethylation levels (Figure 8A), implying that entinostat might induce miR-203 in NSCLC cells via demethylation of *miR-203* promoter. DNA methylation is primarily controlled by a group of enzymes called DNA methyltransferases (DNMTs), among which only DNMT1, DNMT3A, and DNMT3B possess catalytic activity [38, 39]. While DNMT3A and DNMT3B are *de novo* enzymes methylating previously unmethylated DNA, DNMT1 is mainly used to maintain DNA methylation status [40]. It has been shown that DNMT1 stability is regulated by a mechanism involving in acetylation-driven ubiquitination [41], and HDAC inhibition promotes ubiquitin-dependent degradation of DNMT1 in breast cancer cells [42]. We therefore investigated the effects of entinostat on DNMTs in A549 and H460 cells, and discovered that entinostat reduced the protein and mRNA levels of DNMT1, but not DNMT3A and DNMT3B, in a time-dependent manner (Figure 8B–8D). To test if entinostat exhibited a similar activity *in vivo*, we performed western blot analysis using the tumors described in Figure 7. DNMT1 was clearly decreased in the tumors from mice treated with entinostat (not paclitaxel). Strikingly, the combinatorial treatment of entinostat and paclitaxel resulted in most significant reduction of DNMT1 (Figure 9). This additive/synergistic inhibition on DNMT1 was also observed *in vitro* (Supplementary Figure S5). Taken together, our data suggest that entinostat elicits *miR-203* promoter hypomethylation via reduction of DNMT1 *in vitro* and *in vivo*, and thereby enhances expression of this miRNA in NSCLC cells.

### Positive correlation of DNMT1 and Survivin expression is observed in NSCLC samples

**Figure 10:**
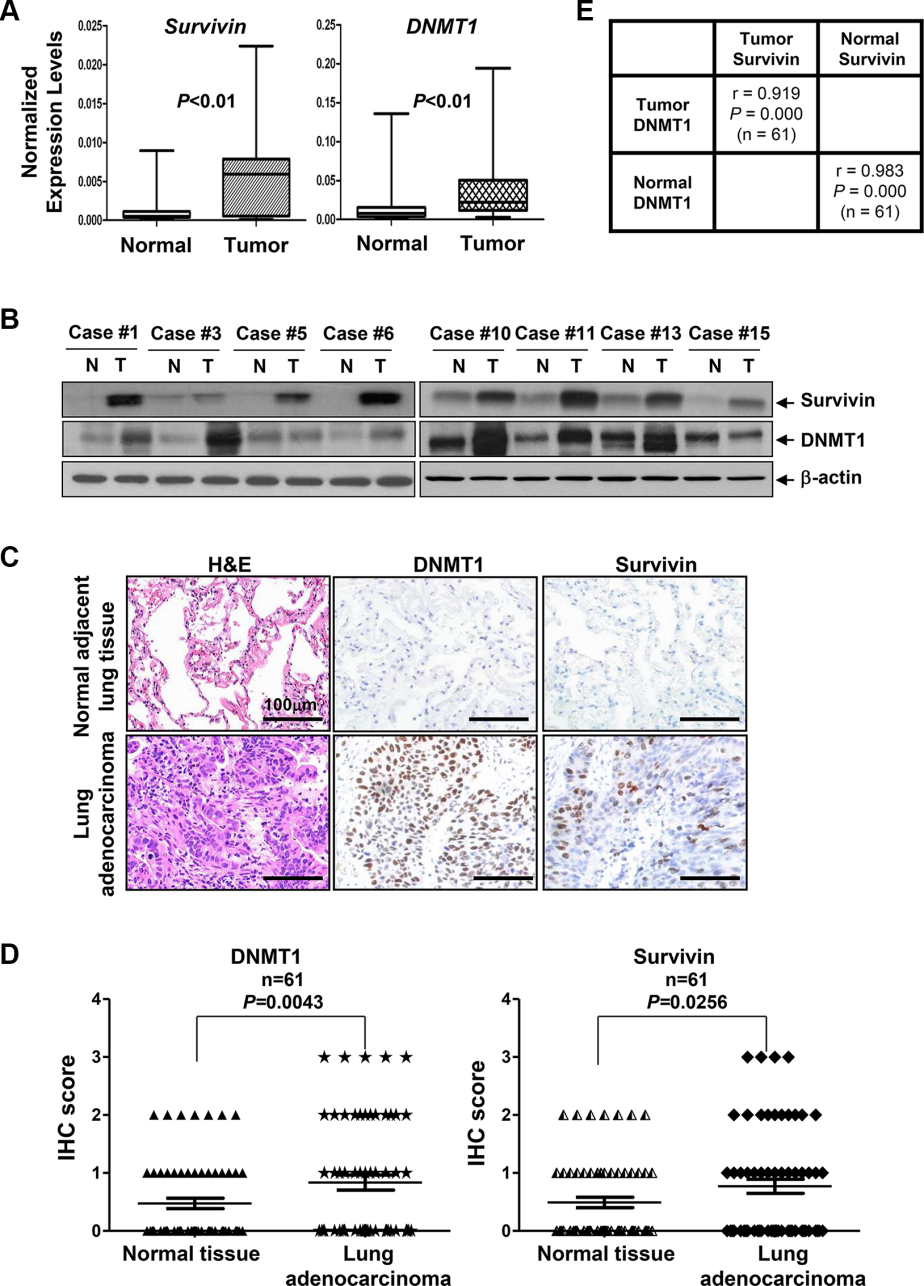
The expression levels of both DNMT1 and Survivin are significantly higher in NSCLC tumors than that in normal lung tissues (**A**) The mRNA expression levels of *Survivin* and *DNMT1* in NSCLC tumors and their self-paired normal adjacent lung tissues (*n* = 20) were measured by qRT-PCR and normalized to *β-actin*. (**B**) Representative western blot analysis of Survivin, DNMT1, or β-actin in NSCLC tumors (T) and their self-paired normal adjacent lung tissues (N). (**C**) Data show the H&E staining and IHC analysis of Survivin and DNMT1 (*n* = 61) in representative lung adenocarcinoma and the self-paired normal adjacent lung tissues. The scale bar represents 100 μm. (**D**) The expression levels of DNMT1 and Survivin in lung adenocarcinoma and their self-paired normal adjacent lung tissues (*n* = 61) were determined by IHC assays. The slides were evaluated by two independent pathologists and scored according to the percentage cells with positive staining for each antigen (0: 0~4%; 1: 5%~25%; 2: 26%~50%; and 3: ≥ 51%). The IHC scores were compared between lung adenocarcinoma and normal lung tissues and statistically analyzed. (**E**) A positive correlation between DNMT1 and Survivin in both lung adenocarcinoma (Tumor) and their self-paired normal adjacent lung tissues (Normal) was observed. Pearson's correlation coefficient (*r*) and *P* values are shown.

Since intrinsic resistance to paclitaxel frequently occurs and causes treatment failure in NSCLC [16], it is conceivable to hypothesize that overexpression of DNMT1 may lead to epigenetic silencing of miRNAs to upregulate Survivin, and thereby compromise the efficacy of paclitaxel in NSCLC patients. To test this hypothesis, we first examined the expression of *miR-203* and *miR-542-3p* as well as *Survivin* and *DNMT1* in 20 freshly-obtained NSCLC samples and their self-paired normal adjacent lung tissues (Table 1). We noticed that the mRNA levels of both *Survivin* and *DNMT1* were significantly elevated in NSCLC as compared to normal lung tissues (Figure 10A). Independent-samples *t*-tests revealed that the means of relative mRNA values of *Survivin* and *DNMT1* in tumors were 8.73-fold and 3.85-fold higher than that of normal tissues, respectively (Table 2). Increased protein levels of Survivin and DNMT1 were also found in 8 of 20 NSCLC samples as compared to normal lung tissues (Figure 10B). To confirm the positive correlation between Survivin and DNMT1, we then retrospectively examined the expression of DNMT1 and Survivin in a set of tissue sections consisting of 61 patients with lung adenocarcinoma. We observed that the staining rates for DNMT1 in NSCLCs and their self-paired normal adjacent lung tissues were 47.5% (29/61) and 36% (22/61), respectively. The staining rates for Survivin in NSCLCs and normal lung tissues were 47.5% (29/61) and 37.7% (23/61), respectively (Supplementary Table S4). Consistent with the western blot data, elevated expression of DNMT1 and Survivin in NSCLC tumors was supported by IHC analyses (Figure 10C). Evaluation of the IHC scores showed that expression of both DNMT1 and Survivin was significantly higher in lung adenocarcinoma than that in normal lung tissues (Figure 10D). The positive correlation between DNMT1 and Survivin was highly significant in both tumors (*r* = 0.919, *P* = 0.000) and normal tissues (*r* = 0.983, *P* = 0.000) (Figure 10E). Interestingly, the expression levels of DNMT1 were shown to be significantly correlated with TNM stages as well as age of the patients (*r* = 1, *P* < 0.0001 by Spearman rank test), while weakly correlated with gender of the patients (*r* = 0.30175, *P* = 0.0181 by Spearman rank test). As such, a strong correlation between the expression of Survivin and TNM stages, but not age or gender of the patients were also revealed by Spearman rank test (Supplementary Table S6). Overall, our clinical analyses suggest that elevated expression of DNMT1 significantly correlates with increased Survivin in NSCLC, especially lung adenocarcinoma.

**Table 1:** The relative mRNA abundance of *miR-203*, *miR-542-3p*, *Survivin*, and *DNMT1*, as well as the methylation status of *miR-203* promoter in the clinical samples of NSCLC patients

Patient Case #	miR-203 (C vs. N)	miR-203 methylation	miR-542-3p (C vs. N)	Survivin (C vs. N)	DNMT1 (C vs. N)
***1***	0.24↓	H (1)	2.04	13.52↑	3.11↑
***2***	0.48	/ (0)	0.36	10.19↑	2.71↑
***3***	0.11↓	H (1)	0.93	3.72↑	7.27↑
***4***	1.02	/ (0)	1.39	0.80	1.00
***5***	0.04↓	H (1)	0.94	8.33↑	5.37↑
***6***	0.25↓	H (1)	0.07↓	33.20↑	7.64↑
***7***	1.07	/ (0)	1.43	1.04	5.40↑
***8***	1.81	/ (0)	0.83	0.74	1.47
***9***	1.57	/ (0)	0.04↓	0.72	0.48
***10***	0.07↓	H (1)	0.54	16.98↑	2.29
***11***	0.12↓	H (1)	0.83	16.85↑	9.78↑
***12***	0.45	/ (0)	0.01↓	1.41	5.78↑
***13***	0.07↓	H (1)	0.30	10.15↑	5.07↑
***14***	0.78	/ (0)	0.42	2.00	1.43
***15***	0.03↓	H (1)	0.24↓	42.52↑	5.20↑
***16***	1.95	/ (0)	0.71	0.66	1.29
***17***	0.85	/ (0)	2.21	0.46	0.44
***18***	0.40	H (1)	0.02↓	1.41	0.23↓
***19***	1.54	H (1)	1.82	1.61	5.63↑
***20***	0.11↓	H (1)	0.24↓	8.33↑	5.37↑

**Notes:** C *vs.* N (cancer *vs.* normal adjacent tissue). The relative mRNA abundance of *miR-203, miR-542-3p*, *Survivin*, or *DNMT1* was presented as 2^^−ΔΔCt^. All data shown were the mean ± SD of three independent experiments. ↓: 2^^−ΔΔCt^ ≤ 0.25 was taken as downregulated; ↑: 2^^−ΔΔC^ ≥ 2.5 was taken as upregulated; and 0.25 < 2^^−ΔΔC^ < 2.5 was taken as unaltered. H: hypermethylation status (scored as 1);

/: no significant difference between cancer and normal tissue (scored as 0).

**Table 2:** Statistics of the relative expression values of *miR-203*, *miR-542-3p*, *Survivin*, and *DNMT1* in the self-paired clinical samples from patients with NSCLC

Group	n	Mean	SD	P value
*miR-203 in normal tissues*	20	1	0	/
*miR-203 in tumors*	20	0.65	0.65	0.023981
*miR-542-3p in normal tissues*	20	1	0	/
*miR-542-3p in tumors*	20	0.77	0.69	0.146602
*Survivin in normal tissues*	20	1	0	/
*Survivin in tumors*	20	8.73	11.50	0.007259
*DNMT1 in normal tissues*	20	1	0	/
*DNMT1 in tumors*	20	3.85	2.76	0.000191

**Notes:** Average value for the miRNA or mRNA abundance in self-paired normal adjacent lung tissues was taken as 1 and the relative miRNA or mRNA abundance in NSCLC tumors was presented as 2^^−ΔΔCt^.

### Hypermethylation of *miR-203* promoter is associated with lower levels of miR-203 and higher levels of both DNMT1 and Survivin in NSCLC tumors

**Figure 11:**
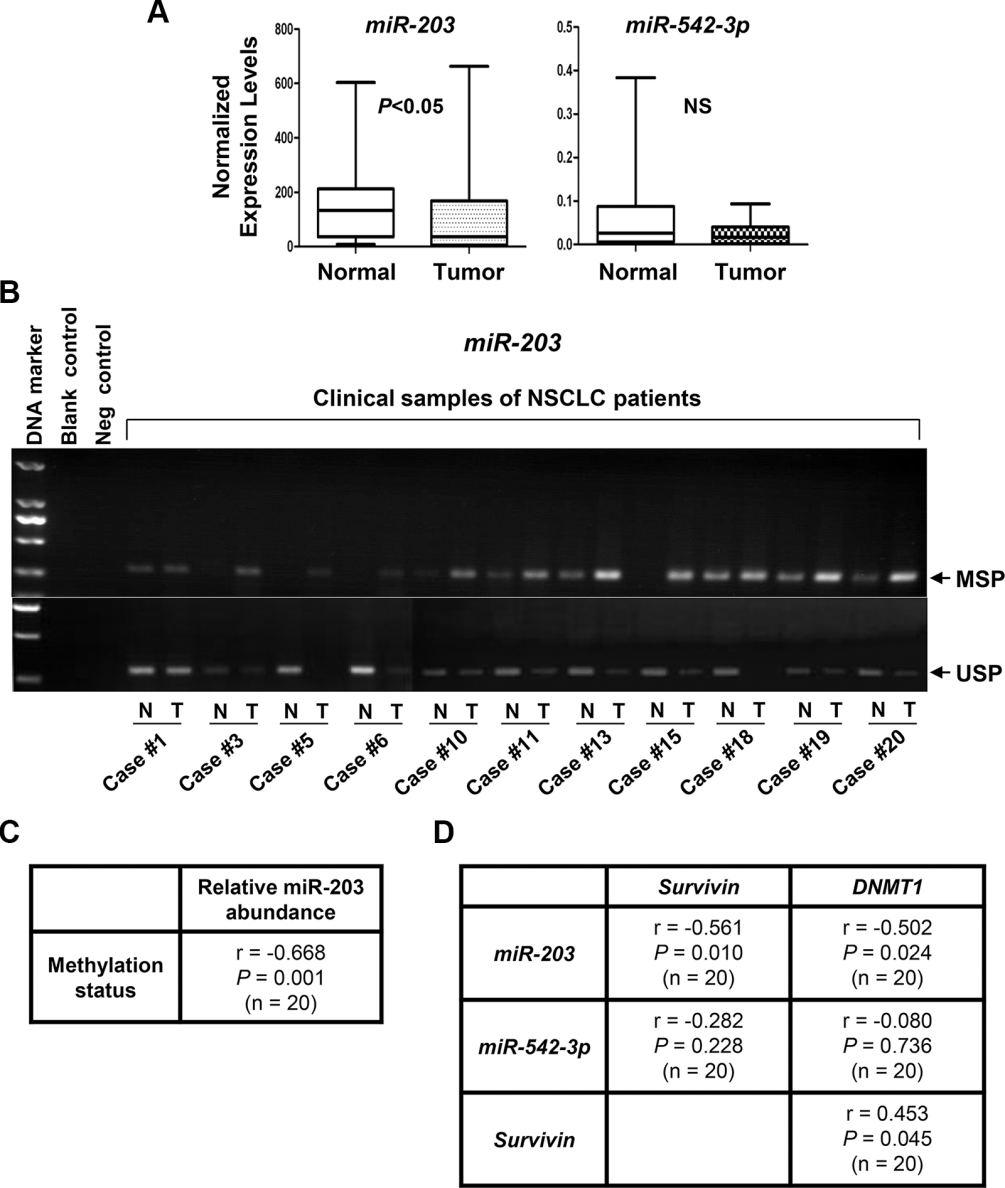
The expression levels of *miR-203*, but not *miR-542-3p*, are significantly increased, associated with *miR-203* promoter hypermethylation, and inversely correlated with the expression of *DNMT1* and *Survivin* in NSCLC tumors (**A**) The expression levels of *miR-203* and *miR-542-3p* in NSCLC tumors and their self-paired normal adjacent lung tissues (*n* = 20) were measured by qRT-PCR and normalized to RNU6B. (**B**) NSCLC tumors (T) and their self-paired normal adjacent lung tissues (N) were subjected to DNA extraction, and followed by methylation-specific PCR (MSP) and unmethylation-specific PCR (USP) analyses of *miR-203* promoter. Representative results are shown. Blank control, double distilled H_2_O as PCR template. Negative control, unconverted genomic DNA of normal lung tissue as PCR template. (**C**) Correlation analysis of methylation status of *miR-203* promoter and its expression levels in NSCLC tumors (*n* = 20) was performed. Pearson's correlation coefficient (r) and *P* value are shown. (**D**) Correlation analyses of *miR-203* or *miR-542-3p* expression with *Survivin* or *DNMT1* expression in NSCLC tumors (*n* = 20) were performed. Pearson's correlation coefficient (r) and *P* values are shown.

We next focused our clinical studies on miR-203 and miR-542-3p. QRT-PCR analyses found that the expression levels of miR-203, but not miR-542-3p were significantly decreased in NSCLCs as compared to that in normal lung tissues (Figure 11A). To explore if miR-203 reduction might be attributed to its promoter methylation, we examined the methylation status of *miR-203* promoter in the clinical samples. The results of MSP and/or USP were summarized in Table 1 and partially shown (Figure 11B). Hypermethylation of *miR-203* promoter was discovered in 11 of 20 NSCLC tumors. The hypermethylation was inversely correlated with the levels of miR-203 in NSCLC (*r* = −0.668, *P* = 0.001; Figure 11C). Independent-samples *t*-test showed that the expression of miR-203, but not miR-542-3p, was significantly reduced in NSCLCs as compared to that in normal lung tissues (Table 2). More importantly, a significant inverse correlation was detected between miR-203 (not miR-542-3p) and the expression of *Survivin* (*r* = −0.561, *P* = 0.01) or *DNMT1* (*r* = −0.502, *P* = 0.024). In contrast, a positive correlation between *Survivin* and *DNMT1* (*r* = 0.453, *P* = 0.045) was observed (Figure 11D), consistent with our IHC analysis of Survivin and DNMT1 in lung adenocarcinoma (Figure 10E). Collectively, our data demonstrate that elevated expression of DNMT1 may give rise to *miR-203* promoter hypermethylation, and thereby lead to Survivin overexpression in NSCLC.

## DISCUSSION

**Figure 12:**
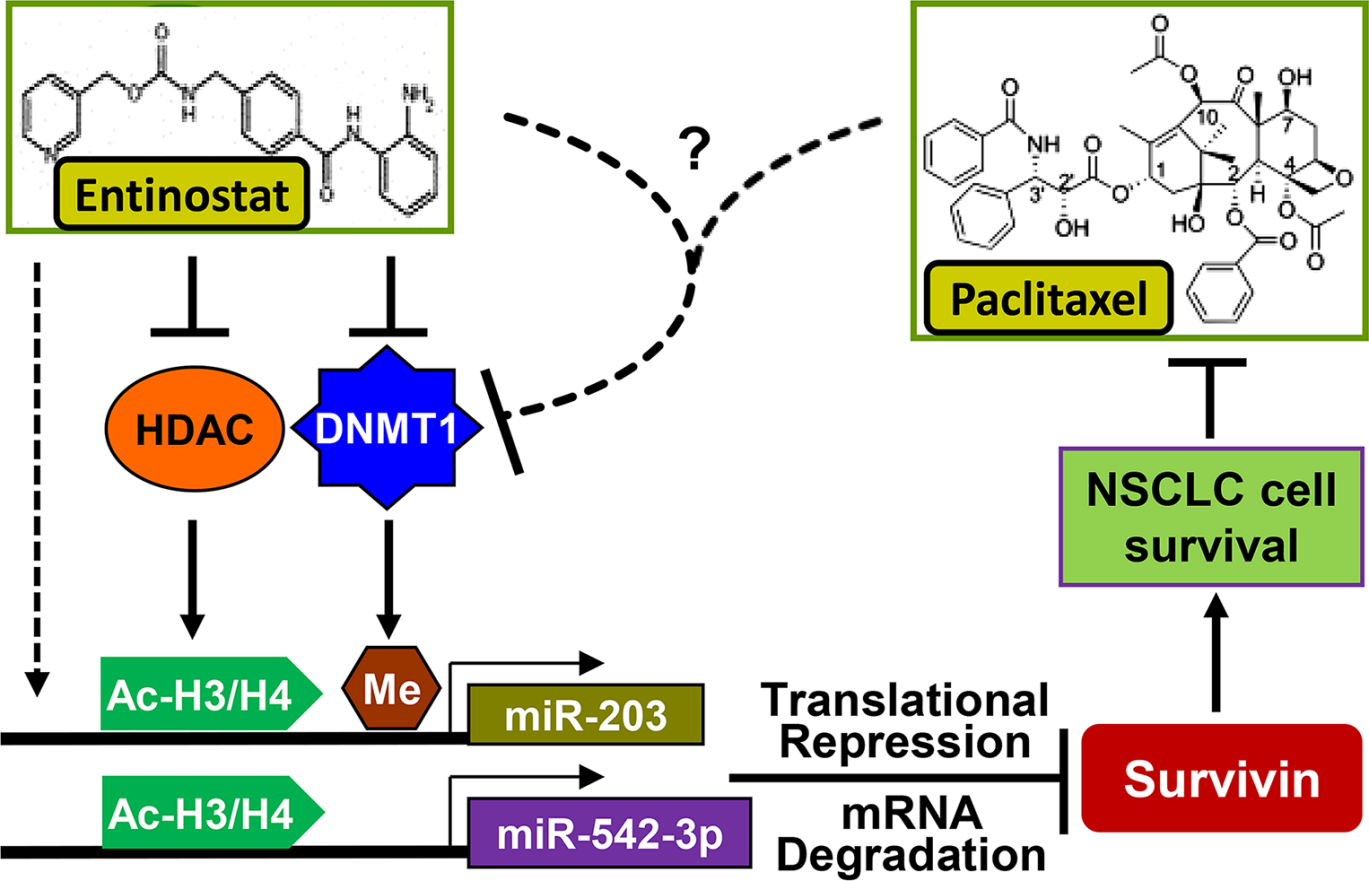
Proposed model underlying the mechanism of entinostat potentiation of paclitaxel-mediated antitumor activity against NSCLC Entinostat enhances expression of miR-203 (*in vitro* and *in vivo*) and miR-542-3p (*in vitro*) via inhibition of HDAC, downregulation of DNMT1, and/or other mechanisms to reduce Survivin, and thereby potentiate paclitaxel-induced apoptosis in NSCLC cells. The combinations of entinostat and paclitaxel exert potent antitumor activity against NSCLC likely due to dramatic reduction of DNMT1 through an unknown mechanism to release promoter methylation-mediated epigenetic silencing of *miR-203*.

Paclitaxel has long been used to treat NSCLC either as single agent or combined with other therapeutics [14, 15]. However, resistance to paclitaxel remains one of the main causes of treatment failure in NSCLC [16]. It is of particular significance to elucidate the underlying mechanism of paclitaxel resistance and identify new strategy to abrogate it. Here, we showed that epigenetic silencing of *miR-203*, leading to Survivin overexpression, played a key role in determining the sensitivity of NSCLC cells to paclitaxel. The HDACi entinostat selectively reduced Survivin via induction of miR-203 and miR-542-3p, and thereby significantly enhanced paclitaxel-induced apoptosis in NSCLC cells. However, induction of miR-542-3p by entinostat was observed *in vitro*, not *in vivo*. Clinical analysis found no difference of miR-542-3p levels between NSCLC tumors and the normal lung tissues. There was no correlation between the expression of miR-542-3p and mRNA levels of either *Survivin* or *DNMT1* (Figure 11A and 11D). These data suggest that 1) increased DNMT1 may not alter miR- 542-3p expression in NSCLC; 2) although miR-542-3p contributes to entinostat-induced growth inhibition and apoptosis *in vitro*, it has little effect on entinostat potentiation of paclitaxel efficacy *in vivo*. Collectively, our studies indicate that inhibition of DNMT1 is at least one of the major mechanisms for entinostat to lift epigenetic silencing of *miR-203*, which results in downregulation of Survivin and thereby enhances paclitaxel-mediated antitumor activity against NSCLC (Figure 12).

Our data is supported by a recent report showing that the pan-HDACi vorinostat or panobinostat significantly promoted cisplatin-induced apoptosis in A549 and H460 cells [43]. Further studies revealed that inhibition of HDAC 1, 2 and 3 with entinostat potentiated cisplatin-induced apoptosis in A549 cells [43]. Our conclusions seemed to be different from the findings of another report [44], which showed that epigenetic therapy (azacitidine plus entinostat) did not alter selected NSCLC cells− responsiveness to cisplatin, docetaxel, gemcitabine, and vinorelbine *in vitro* and *in vivo*. In addition to the fact that paclitaxel was not tested in the later study [44], this disparity might be explained by the distinct treatment regimens used. 1) We and Riley *et al.* [43] combined entinostat with chemotherapy (paclitaxel in our study and cisplatin in Riley's) simultaneously, whereas sequential treatment - epigenetic therapy followed by chemotherapy - was evaluated by Vendetti *et al.* [44]. 2) While epigenetic therapy consisting of both azacitidine and entinostat was used by Vendetti *et al.* only entinostat was applied in our study and Riley's. Thus, the doses of entinostat in Vendetii's study were much lower than that used in ours and Riley's. Nonetheless, the doses of entinostat we used are still within the clinically-relevant range [21]. Although the data by Vendetti [44] call into question whether epigenetic agents could potentiate chemotherapy in cancer treatment, it is possible that their models and sequential treatments do not fully reflect the genetic and/or epigenetic alterations in a subset of NSCLC patients who may benefit from epigenetic priming. Our data support that further evaluation in both preclinical models and clinical trials to test the therapeutic potential of entinostat in combination with paclitaxel against NSCLC is warranted.

Elevated expression of Survivin is observed in almost all types of human malignancies and positively correlates with poor prognosis, tumor recurrence, and drug resistance [9, 10]. However, the molecular mechanisms controlling Survivin expression in cancers have not been fully elucidated. Recent studies show that a number of miRNAs, including miR-203 and miR-542-3p, play critical roles in regulating Survivin expression in cancer cells [45]. Nonetheless, majority of the studies are carried out in preclinical models, it is currently unknown whether the expression of miR-203 and/or miR-542-3p is altered in NSCLC tumors. Our analysis suggests that miR-203 is frequentlydownregulated due to promoter methylation in NSCLC, and the reduced miR-203 is inversely correlated with the expression of *Survivin* and *DNMT1* (Figure 11D). These findings support the importance of specific miRNA in regulation of Survivin expression. Thus, it is conceivable to hypothesize that novel therapies which are able to induce expression of miR-203 will effectively downregulate Survivin, and therefore sensitize the cancer cells to paclitaxel treatment. Our data presented in this manuscript with entinostat to enhance miR-203 expression strongly support this notion. Our studies with the second set of clinical samples (Figure 10 and Supplementary Table S5 & S6) seem to suggest that there is a significant correlation between DNMT1 and Survivin in adenocarcinoma. However, after careful analysis of all of our clinical data, we could not rule out the possibility that this correlation may also occur in squamous cell carcinoma. Our first set of clinical samples (20 freshly-obtained NSCLC samples) contains 10 squamous cell carcinomas (Figure 11D, Table 1 and Supplementary Table S3). Among these squamous cell carcinomas, increased mRNA expression of both *DNMT1* and *Survivin* has been shown in 4 of them (#6, #11, #15, and #20). Thus, to determine whether the correlation of DNMT1 and Survivin may have preference towards certain histology subtypes of NSCLC, we need to expand the population of patients with all subtypes in our future work.

DNA methylation has long been recognized as a key player in tumor initiation and progression [46]. Yet, epigenetic and genetic mechanisms often intertwine and take advantage of each other during tumorigenesis [47]. Our discovery that increased DNMT1 negatively correlates with miR-203 expression in NSCLC offers a representative paradigm regarding the cross-talk between genetic and epigenetic alterations. As a global enzyme maintaining DNA methylation status, how does the dysregulated DNMT1 link to specific silencing of *miR-203* in NSCLC is an unsolved question. A recent study reveals that a long noncoding RNA termed *ecCEBPA* is critical for regulation of DNA methylation at a particular site via interaction with DNMT1 [48]. Whether a similar mechanism accounts for *miR-203* promoter methylation in NSCLC by DNMT1 awaits further investigation.

In summary, we demonstrate that entinostat downregulates Survivin via induction of miR-203 and miR-542-3p *in vitro* and/or *in vivo*, and thereby potentiates paclitaxel-mediated antitumor activity against NSCLC. Clinical studies show that increased DNMT1 positively correlates with *miR-203* promoter methylation and overexpression of Survivin in NSCLC tumors. These findings shed new lights on the underlying mechanism of Survivin upregulation in NSCLC. Our data suggest that the addition of entinostat to paclitaxel-based regimens in selected NSCLC patients with increased DNMT1 and/or Survivin may exhibit significant survival benefit.

## MATERIALS AND METHODS

### Reagents and antibodies

Paclitaxel was obtained from University of Colorado Hospital pharmacy. Entinostat was from LC Laboratories (Woburn, MA). The miRNA mimics and negative controls were from Thermo Scientific Dharmacon (Lafayette, CO). The lentiviral pLKO.1 system containing shRNA was described previously [17, 19]. The lentiviral miRZip^™^ system (Scramble control, miRZip-203, miRZip-542-3p) were products of System Biosciences, Inc. (Mountain View, CA). Antibodies were obtained as follows: Survivin (6E4) mouse mAb, Histone 3, Acetyl-Histone H3 (Lys9), mTOR, P-mTOR (Ser 2448), caspase-8, caspase-3, cleaved caspase-3, PARP, P-MAPK (Erk1/2), MAPK (Erk1/2), P-Akt (Ser473), Akt, and Bcl-xL (Cell Signaling Technology, Inc., Beverly, MA); Mcl-1 (BD Biosciences, San Jose, CA); DNMT1, DNMT3A, and DNMT3B (Epitomics, Burlingame, CA); Ki-67 (Thermo Fisher Scientific Inc., Waltham, MA); b-actin (Sigma Co., St. Louis, MO). All other reagents were from Sigma unless otherwise specified.

### Cells and cell culture

NSCLC cell lines A549 and H460 were from ATCC (Manassas, VA) and maintained in RPMI1640 medium supplemented with 10% fetal bovine serum (FBS). HEK293T human embryonic kidney cells were maintained in DMEM/F12 medium containing 10% FBS. The cells were free of mycoplasma contamination, determined by the MycoAlert™ Mycoplasma Detection Kit (Lonza Group Ltd. Basel, Switzerland) every three months. All cell lines were cultured in a 37°C humidified atmosphere containing 95% air and 5% CO_2_ and were split twice a week.

### Cell proliferation, western blots, apoptosis, and quantitative real-time PCR (qRT-PCR)

The CellTiter96AQ cell proliferation kit (Thermo Fisher Scientific) to determine cell viability, western blots to examine protein expression and activation, an apoptosis ELISA kit (Roche Diagnostics Corp., Indianapolis, IN) to measure histone-associated DNA fragments, and qRT-PCR with the FastStart Universal SYBR Green Master Mixes (Roche) by a Real-Time PCR system (Applied Biosystems, Foster City, CA) were described previously [17, 18, 20].

### Clonogenic assay

Clonogenic assays were performed as described previously [49, 50]. The colony was defined to consisting of at least 50 cells. The colony numbers were quantified with QuantiOne software of Fluor-S^™^ Multimager (Bio-Rad Laboratories, Inc., Hercules, CA).

### Construction of lentiviral expression vector pLEX-hSurvivin

The coding sequence of human *Survivin* was amplified from pcDNA3-*hSurvivin* (provided by Dr. Dario Altieri at The Wistar Institute Cancer Center) by PCR with the following forward primer: 5**′**-ATA GCG GCC GCA TGG GTG CCC CGA CGT TGC-3**′** and reverse primer: 5**′**-GCG ACG CGT TCA ATC CAT GGC AGC CAG CTG-3**′**. The amplified fragments were inserted into the lentiviral vector pLEX-MCS (Open Biosystem, Huntsville, AL). After sequencing verification, the recombinant was nominated as pLEX-*hSurvivin*.

### Production of lentivirus

Lentiviral production was performed as described [17, 51]. In brief, the lentiviral expression vector pLKO.1-ConshRNA or pLKO.1-SurshRNA; pLEX-Luc or pLEX-*hSurvivin*; miRZip control, miRZip-203, or miRZip-542-3p and the packaging plasmids psPAX2 and pMD2.G were co-transfected into HEK293T cells. After 24 h, the culture media were changed with fresh media. The virus-containing media were collected, aliquot and stored at −80°C.

### Specific inhibition of miRNA expression with a lentiviral system

Lentiviral miRZip expression system (scramble control, miRZip-203, or miRZip-542-3p) was applied to fulfill specific inhibition of miR-203 or miR-542-3p in NSCLC cells.

### Analysis of miRNA expression

The expression of mature miR-203, miR-542-3p, Let-7c, miR-29b, and RNU6B was measured by RT-PCR using TaqMan Assays (Applied Biosystems) as described previously [20]. The relative miRNA levels were calculated using the comparative Ct method (ΔΔCt).

### Tumor xenograft model

Athymic nu/nu mice (Shanghai SLAC Laboratory Animal Co. Ltd., Shanghai, China) were maintained in accordance with the IACUC procedures and guidelines. Five × 10^6^ A549-Fluc or H460-Fluc cells were suspended in 100 μL of PBS, mixed with Matrigel (BD Biosciences) and injected subcutaneously into the flanks of 5-week-old female mice. Tumor formation was assessed by palpation and measured with fine calipers three times a week. Tumor volume was calculated by the formula: *Volume* = (*Length* × *Width*^2^)/2, which was statistically analyzed as we described previously [18, 52]. When tumors reach ~150 mm^3^, mice were randomly assigned into four groups (*n* = 4 and *n* = 3 for A549-Fluc and H460-Fluc cells, respectively): 1) control mice received intraperitoneal (i.p.) injection of 100 μl of PBS; 2) mice received i.p. injection of entinostat (25 mg/kg for A549-Fluc or 12.5 mg/kg for H460-Fluc) in 100 μl PBS twice a week; 3) mice received i.p. injection of paclitaxel (7.5 mg/kg) in 100 μl PBS twice a week; 4) mice received i.p. injection of entinostat and paclitaxel in 100 μl PBS twice a week. At the end of study, mice were euthanized according to approved protocol. Tumors were excised and subjected to immunohistochemistry analyses and qRT-PCR measurement of miRNA expression.

### Bioluminescence imaging

The IVIS200 instrument (Caliper Life Sciences, PerkinElmer, Waltham, MA) was used. For cultured cells, we imaged luciferase signal by adding PBS or d-luciferin at a concentration of 0.15 mg/mL. For monitoring luciferase-labeled tumor cells *in vivo*, mice to be imaged were i.p. injected with 150 mg/kg body weight of d-luciferin in 200 μl of PBS and then anesthetized with continuous flow of isofluorane. Imaging of the mice was carried out 10 min later. All images were processed by manufacturer-supplied software for quantitative analysis.

### Immunohistochemistry (IHC)

IHC was performed as described previously [18, 52]. The specificity of all antibodies - Ki-67 (rabbit monoclonal SP6; dilution 1:500 in TBST + 1% BSA), cleaved caspase-3 (rabbit polyclonal; 1:1,000), Survivin (Epitomics; rabbit monoclonal EP2880Y; 1:100), DNMT1 (Epitomics; rabbit monoclonal EPR3521-2; 1:300) - has been confirmed by both positive and negative controls. Antibody complexes were visualized with IP Flex DAB (Biocare). All sections were counterstained in Mayer's hematoxylin, nuclei blued in 1% ammonium hydroxide, cleared in xylene and cover glass mounted by synthetic resin.

### Methylation-specific PCR (MSP)

MSP for DNA methylation was performed as described by Chim *et al.* [36]. Treatment of DNA with bisulfite for conversion of unmethylated cytosine to uracil was performed with the EpiTect Bisulfite Kit (Qiagen). Primers used for MSP and unmethylation-specific PCR (USP) were shown in Supplementary Table S1. PCR products were loaded onto 1.5% agarose gels and electrophoresed. The images were obtained by exposure of the gels to an ultraviolet trans-illuminator and recorded with Fluor-S^™^ Multimager (Bio-Rad Laboratories).

### Statistical analysis

All results were confirmed by at least three independent experiments. Data are presented as mean ± SD. Statistical analysis was performed using SPSS 19.0 software (SPSS Institute). Student's *t* tests were used for comparisons of means of quantitative data between groups. The correlations between *miR-203* promoter methylation and miR-203 expression, between miR-203 and Survivin expression, and between DNMT1 and Survivin expression were evaluated by Pearson's correlation coefficient (r). Values of *P* < 0.05 were considered significant.

### Study approval

Animal experimental procedures were approved by the Institutional Animal Care and Use Committee at Fuzhou General Hospital of Xiamen University. The clinical samples of NSCLC patients were acquired with informed consent following protocols approved by Fuzhou General Hospital of Xiamen University Research Ethics Board. The patients' clinical characteristics are summarized in Supplementary Tables S2–S5.

## SUPPLEMENTARY MATERIALS FIGURES AND TABLES



## Abbreviations

NSCLC: non-small cell lung cancer
IAP: inhibitor of apoptosis
HDAC: histone deacetylase
DNMT: DNA methyltransferase
PI-3K: phosphoinositide 3-kinase
PARP: poly (ADP-ribose) polymerase
MSP: Methylation-specific PCR
USP: unmethylation-specific PCR
qRT-PCR: quantitative real-time PCR
IHC: immunohistochemistry
ELISA: enzyme-linked immunosorbent assay
MTS: 3-(45-dimethylthiazol-2-yl)-5-(3-carboxymethoxyphenyl)-2-(4-sulfophenyl)-2H-tetrazolium inner salt

## References

[1] Jemal A, Bray F, Center MM, Ferlay J, Ward E, Forman D (2011). Global cancer statistics. CA Cancer J Clin.

[2] Siegel R, Ma J, Zou Z, Jemal A (2014). Cancer statistics, 2014. CA Cancer J Clin.

[3] Wu YK, Huang CY, Yang MC, Lan CC, Lee CH, Chan EC, Chen KT (2014). Nuclear survivin expression: a prognostic factor for the response to taxane-platinum chemotherapy in patients with advanced non-small cell lung cancer. Med Oncol.

[4] Pao W, Chmielecki J (2010). Rational, biologically based treatment of EGFR-mutant non-small-cell lung cancer. Nat Rev Cancer.

[5] Langer CJ, Besse B, Gualberto A, Brambilla E, Soria JC (2010). The evolving role of histology in the management of advanced non-small-cell lung cancer. J Clin Oncol.

[6] Ettinger DS, Akerley W, Borghaei H, Chang AC, Cheney RT, Chirieac LR, D'Amico TA, Demmy TL, Govindan R, Grannis FW, Grant SC, Horn L, Jahan TM (2013). Non-small cell lung cancer, version 2.2013. J Natl Compr Canc Netw.

[7] Altieri DC (2008). Survivin, cancer networks and pathway-directed drug discovery. Nat Rev Cancer.

[8] Kanwar JR, Kamalapuram SK, Kanwar RK (2013). Survivin signaling in clinical oncology: a multifaceted dragon. Med Res Rev.

[9] Zaffaroni N, Daidone MG (2002). Survivin expression and resistance to anticancer treatments: perspectives for new therapeutic interventions. Drug Resist Updat.

[10] Coumar MS, Tsai FY, Kanwar JR, Sarvagalla S, Cheung CH (2013). Treat cancers by targeting survivin: just a dream or future reality?. Cancer Treat Rev.

[11] Rauch A, Hennig D, Schafer C, Wirth M, Marx C, Heinzel T, Schneider G, Kramer OH (2014). Survivin and YM155: how faithful is the liaison?. Biochimica et biophysica acta.

[12] Kelly RJ, Thomas A, Rajan A, Chun G, Lopez-Chavez A, Szabo E, Spencer S, Carter CA, Guha U, Khozin S, Poondru S, Van Sant C, Keating A (2013). A phase I/II study of sepantronium bromide (YM155, survivin suppressor) with paclitaxel and carboplatin in patients with advanced non-small-cell lung cancer. Ann Oncol.

[13] Schiller JH, Harrington D, Belani CP, Langer C, Sandler A, Krook J, Zhu J, Johnson DH (2002). Comparison of four chemotherapy regimens for advanced non-small-cell lung cancer. N Engl J Med.

[14] Sandler A, Gray R, Perry MC, Brahmer J, Schiller JH, Dowlati A, Lilenbaum R, Johnson DH (2006). Paclitaxel-carboplatin alone or with bevacizumab for non-small-cell lung cancer. N Engl J Med.

[15] Socinski MA (1999). Single-agent paclitaxel in the treatment of advanced non-small cell lung cancer. Oncologist.

[16] Orr GA, Verdier-Pinard P, McDaid H, Horwitz SB (2003). Mechanisms of Taxol resistance related to microtubules. Oncogene.

[17] Wang S, Huang X, Lee CK, Liu B (2010). Elevated expression of erbB3 confers paclitaxel resistance in erbB2-overexpressing breast cancer cells via upregulation of Survivin. Oncogene.

[18] Wang S, Huang J, Lyu H, Cai B, Yang X, Li F, Tan J, Edgerton SM, Thor AD, Lee CK, Liu B (2013). Therapeutic targeting of erbB3 with MM-121/SAR256212 enhances antitumor activity of paclitaxel against erbB2-overexpressing breast cancer. Breast Cancer Res.

[19] Huang X, Gao L, Wang S, Lee CK, Ordentlich P, Liu B (2009). HDAC inhibitor SNDX-275 induces apoptosis in erbB2-overexpressing breast cancer cells via down-regulation of erbB3 expression. Cancer Res.

[20] Wang S, Huang J, Lyu H, Lee CK, Tan J, Wang J, Liu B (2013). Functional cooperation of miR-125a, miR-125b, and miR-205 in entinostat-induced downregulation of erbB2/erbB3 and apoptosis in breast cancer cells. Cell Death Dis.

[21] Knipstein J, Gore L (2011). Entinostat for treatment of solid tumors and hematologic malignancies. Expert Opin Investig Drugs.

[22] Oronsky B, Oronsky N, Knox S, Fanger G, Scicinski J (2014). Episensitization: therapeutic tumor resensitization by epigenetic agents: a review and reassessment. Anticancer Agents Med Chem.

[23] Jones PA (2014). At the tipping point for epigenetic therapies in cancer. J Clin Invest.

[24] Huang X, Wang S, Lee CK, Yang X, Liu B (2011). HDAC inhibitor SNDX-275 enhances efficacy of trastuzumab in erbB2-overexpressing breast cancer cells and exhibits potential to overcome trastuzumab resistance. Cancer Lett.

[25] Lee CK, Wang S, Huang X, Ryder J, Liu B (2010). HDAC inhibition synergistically enhances alkylator-induced DNA damage responses and apoptosis in multiple myeloma cells. Cancer Lett.

[26] Bartel DP (2009). MicroRNAs: target recognition and regulatory functions. Cell.

[27] Bian K, Fan J, Zhang X, Yang XW, Zhu HY, Wang L, Sun JY, Meng YL, Cui PC, Cheng SY, Zhang J, Zhao J, Yang AG, Zhang R (2012). MicroRNA-203 leads to G1 phase cell cycle arrest in laryngeal carcinoma cells by directly targeting survivin. FEBS Lett.

[28] Yoon S, Choi YC, Lee S, Jeong Y, Yoon J, Baek K (2010). Induction of growth arrest by miR-542-3p that targets survivin. FEBS Lett.

[29] Mott JL, Kobayashi S, Bronk SF, Gores GJ (2007). mir-29 regulates Mcl-1 protein expression and apoptosis. Oncogene.

[30] Shimizu S, Takehara T, Hikita H, Kodama T, Miyagi T, Hosui A, Tatsumi T, Ishida H, Noda T, Nagano H, Doki Y, Mori M, Hayashi N (2010). The let-7 family of microRNAs inhibits Bcl-xL expression and potentiates sorafenib-induced apoptosis in human hepatocellular carcinoma. J Hepatol.

[31] Boll K, Reiche K, Kasack K, Morbt N, Kretzschmar AK, Tomm JM, Verhaegh G, Schalken J, von Bergen M, Horn F, Hackermuller J (2013). MiR-130a, miR-203 and miR-205 jointly repress key oncogenic pathways and are downregulated in prostate carcinoma. Oncogene.

[32] Craig VJ, Cogliatti SB, Rehrauer H, Wundisch T, Muller A (2011). Epigenetic silencing of microRNA-203 dysregulates ABL1 expression and drives Helicobacter-associated gastric lymphomagenesis. Cancer Res.

[33] Furuta M, Kozaki KI, Tanaka S, Arii S, Imoto I, Inazawa J (2010). miR-124 and miR-203 are epigenetically silenced tumor-suppressive microRNAs in hepatocellular carcinoma. Carcinogenesis.

[34] Zhang Z, Zhang B, Li W, Fu L, Zhu Z, Dong JT (2011). Epigenetic Silencing of miR-203 Upregulates SNAI2 and Contributes to the Invasiveness of Malignant Breast Cancer Cells. Genes Cancer.

[35] Bueno MJ, Perez de Castro I, Gomez de Cedron M, Santos J, Calin GA, Cigudosa JC, Croce CM, Fernandez-Piqueras J, Malumbres M (2008). Genetic and epigenetic silencing of microRNA-203 enhances ABL1 and BCR-ABL1 oncogene expression. Cancer Cell.

[36] Chim CS, Wong KY, Leung CY, Chung LP, Hui PK, Chan SY, Yu L (2011). Epigenetic inactivation of the hsa-miR-203 in haematological malignancies. J Cell Mol Med.

[37] Jin J, Deng J, Wang F, Xia X, Qiu T, Lu W, Li X, Zhang H, Gu X, Liu Y, Cao W, Shao W (2013). The expression and function of microRNA-203 in lung cancer. Tumour Biol.

[38] Veeck J, Esteller M (2010). Breast cancer epigenetics: from DNA methylation to microRNAs. J Mammary Gland Biol Neoplasia.

[39] Taby R, Issa JP (2010). Cancer epigenetics. CA Cancer J Clin.

[40] Jones PA, Liang G (2009). Rethinking how DNA methylation patterns are maintained. Nat Rev Genet.

[41] Du Z, Song J, Wang Y, Zhao Y, Guda K, Yang S, Kao HY, Xu Y, Willis J, Markowitz SD, Sedwick D, Ewing RM, Wang Z (2010). DNMT1 stability is regulated by proteins coordinating deubiquitination and acetylation-driven ubiquitination. Sci Signal.

[42] Zhou Q, Agoston AT, Atadja P, Nelson WG, Davidson NE (2008). Inhibition of histone deacetylases promotes ubiquitin-dependent proteasomal degradation of DNA methyltransferase 1 in human breast cancer cells. Mol Cancer Res.

[43] Riley JS, Hutchinson R, McArt DG, Crawford N, Holohan C, Paul I, Van Schaeybroeck S, Salto-Tellez M, Johnston PG, Fennell DA, Gately K, O'Byrne K, Cummins R (2013). Prognostic and therapeutic relevance of FLIP and procaspase-8 overexpression in non-small cell lung cancer. Cell Death Dis.

[44] Vendetti FP, Topper M, Huang P, Dobromilskaya I, Easwaran H, Wrangle J, Baylin SB, Poirier JT, Rudin CM (2015). Evaluation of azacitidine and entinostat as sensitization agents to cytotoxic chemotherapy in preclinical models of non-small cell lung cancer. Oncotarget.

[45] Huang J, Lyu H, Wang J, Liu B (2015). MicroRNA regulation and therapeutic targeting of survivin in cancer. Am J Cancer Res.

[46] Baylin SB, Jones PA (2011). A decade of exploring the cancer epigenome - biological and translational implications. Nat Rev Cancer.

[47] You JS, Jones PA (2012). Cancer genetics and epigenetics: two sides of the same coin?. Cancer Cell.

[48] Di Ruscio A, Ebralidze AK, Benoukraf T, Amabile G, Goff LA, Terragni J, Figueroa ME, De Figueiredo Pontes LL, Alberich-Jorda M, Zhang P, Wu M, D'Alo F, Melnick A (2013). DNMT1-interacting RNAs block gene-specific DNA methylation. Nature.

[49] Liu B, Fan Z, Edgerton SM, Yang X, Lind SE, Thor AD (2011). Potent anti-proliferative effects of metformin on trastuzumab-resistant breast cancer cells via inhibition of erbB2/IGF-1 receptor interactions. Cell Cycle.

[50] Lyu H, Yang XH, Edgerton SM, Thor AD, Wu X, He Z, Liu B (2016). The erbB3- and IGF-1 receptor-initiated signaling pathways exhibit distinct effects on lapatinib sensitivity against trastuzumab-resistant breast cancer cells. Oncotarget.

[51] Huang J, Lyu H, Wang J, Liu B (2015). Influence of survivin-targeted therapy on chemosensitivity in the treatment of acute myeloid leukemia. Cancer Lett.

[52] Huang J, Wang S, Lyu H, Cai B, Yang X, Wang J, Liu B (2013). The anti-erbB3 antibody MM-121/SAR256212 in combination with trastuzumab exerts potent antitumor activity against trastuzumab-resistant breast cancer cells. Mol Cancer.

