# Supplementary material for: MicroRNA-mediated epigenetic targeting of Survivin significantly enhances the antitumor activity of paclitaxel against non-small cell lung cancer
Source: Oncotarget. 2016 May 10;7(25):37693–713. doi: 10.18632/oncotarget.9264 (PMC5122342; doi:10.18632/oncotarget.9264)
Supplement: Supplementary file 1 [file oncotarget-07-37693-s001.pdf]

MicroRNA-mediated epigenetic targeting of Survivin significantly enhances the antitumor activity of paclitaxel against non-small cell lung cancer

Supplementary Materials

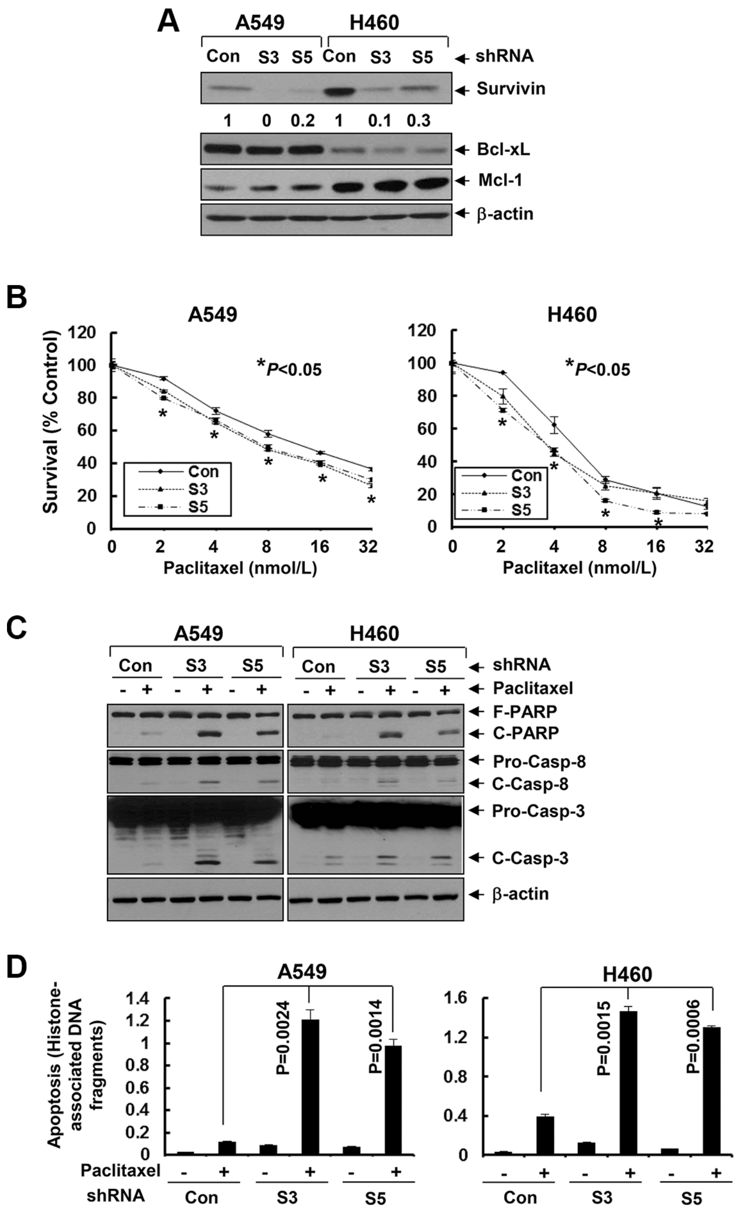

Supplementary Figure S1: Specific knockdown of Survivin expression sensitizes NSCLC cells to paclitaxel-induced growth inhibition and apoptosis. A549 and H460 cells infected with lentivirus containing either control shRNA (Con) or Survivin shRNA (S3 and S5) for 24 h were subjected to the following experiments. (**A**) Western blot analyses of Survivin, Bcl-xL, Mcl-1, or β-actin. The densitometry analyses of Survivin signals were shown underneath. The arbitrary numbers indicate the intensities of each cell line relative to controls, defined as 1.0. (**B)** Cell proliferation (MTS) assays were performed to determine the percentages of surviving cells upon paclitaxel treatment from each cell line relative to controls, defined as 100% survival. Data shows the representative of three independent experiments. *Bars*, S.D. (**C** and **D**) The cells untreated or treated with paclitaxel (6 nmol/L and 3 nmol/L for A549 and H460, respectively) for additional 24 h were collected and subjected to western blot analyses of PARP, caspase-8, caspase-3, or β-actin (C) or apoptotic-ELISA (D).

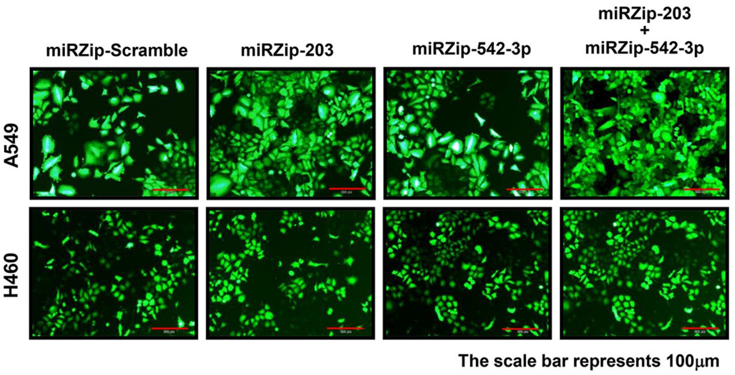

Supplementary Figure S2: Transfection efficiency of A549 and H460 cells with a lentiviral miRZip™ system is determined by GFP expression. A549 and H460 cells were either infected with a GFP-containing lentiviral miRZip™ expression system consisting of either scramble control, or miRZip-203, or miRZip-542-3p, or co-infected with both miRZip-203 and miRZip-542-3p lentiviral miRZip™ expression system. After 24 h, all infected cells were subjected to selection with Puromycin (1 μg/mL) for two weeks and then visualized under a fluorescence microscope.

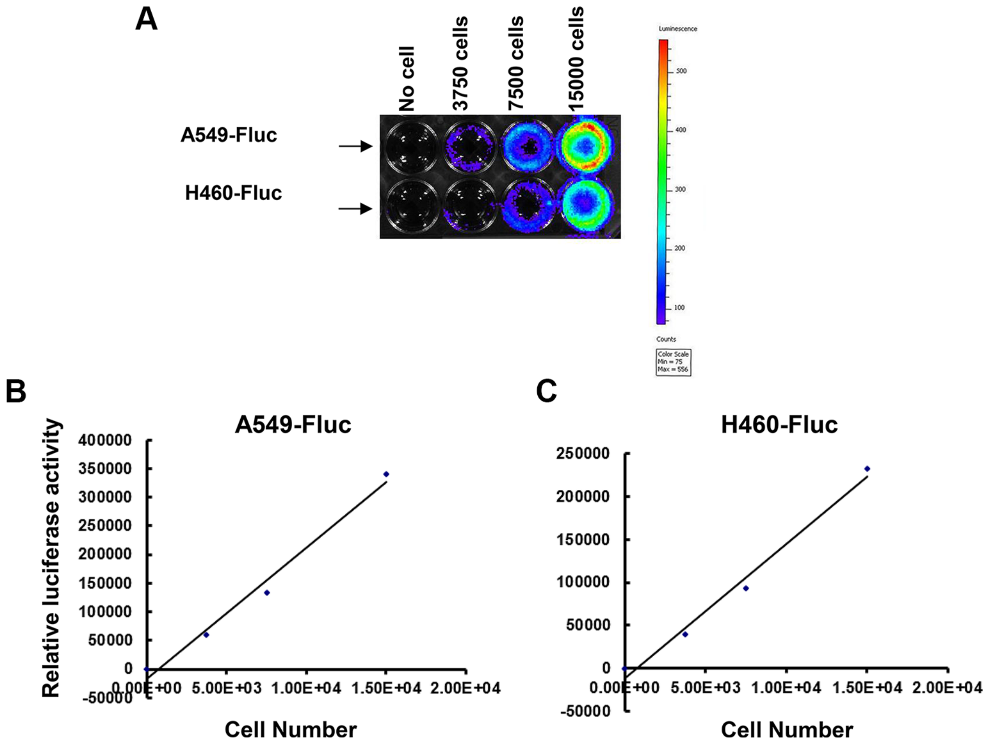

Supplementary Figure S3: A linear correlation between the luciferase activity and seeded numbers of firefly luciferase-labeled A549 (A549-Fluc) or H460 (H460-Fluc) cells is observed. (**A**) Different numbers of A549-Fluc or H460-Fluc cells ranging from 0 to 1.5×10^4 were planted onto 12-well plates for 24 h and then subjected to bioluminescence imaging assay using IVIS200 instrument. (**B** and **C**) The linear correlation between relative luciferase activity and cell numbers was determined by curve fitting analysis.

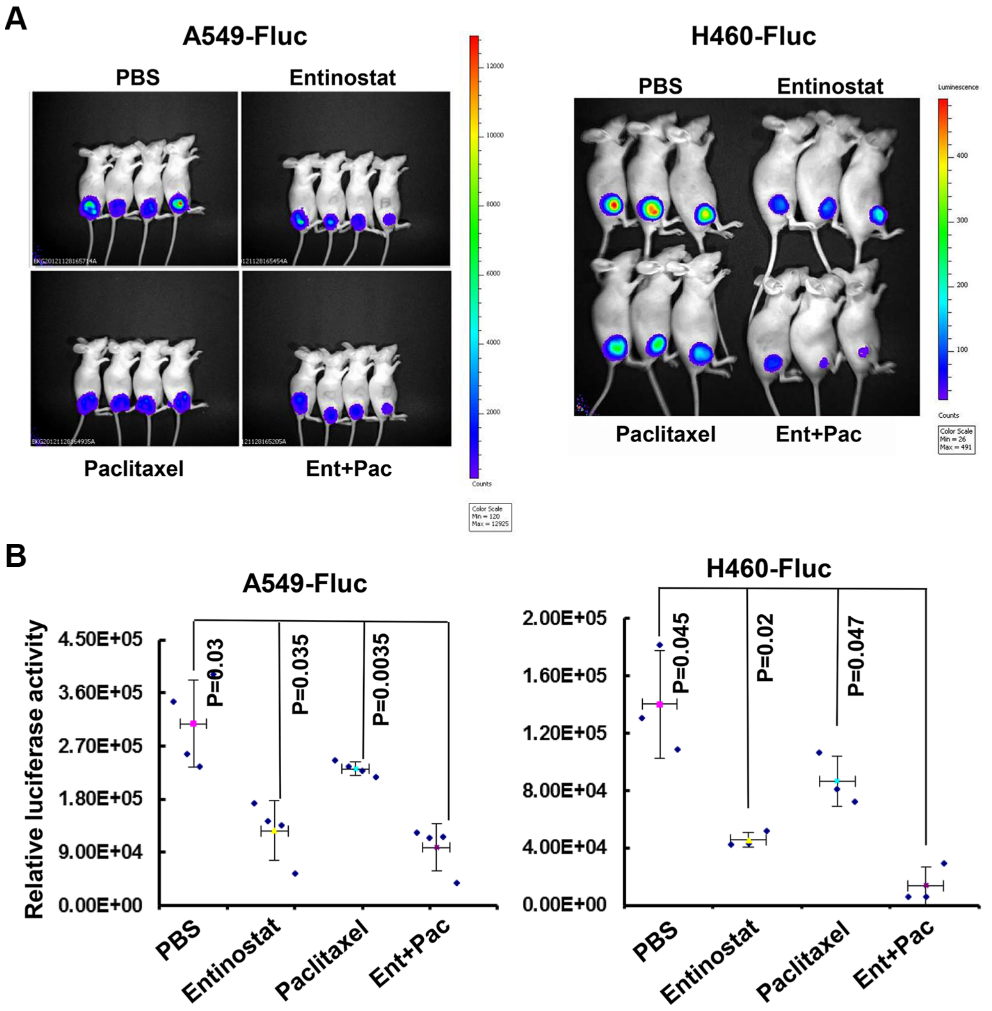

Supplementary Figure S4: *In vivo* tumor growth of NSCLC cells following treatment with entinostat and/or paclitaxel is analyzed by bioluminescence imaging (related to Figure 7). The tumor-bearing mice received intraperitoneal injections of either PBS, or entinostat or paclitaxel alone, or both entinostat and paclitaxel as described in the legends of Figure 7. After five treatments, all mice were subjected to bioluminescence imaging. (**A**) Representative images of tumor-bearing mice were shown. (**B**) The relative luciferase activity in mice treated with combinations of entinostat and paclitaxel was significantly lower than that in mice treated with P BS, or entinostat, or paclitaxel. The luciferase activity was obtained according to the proton reading by IVIS200.

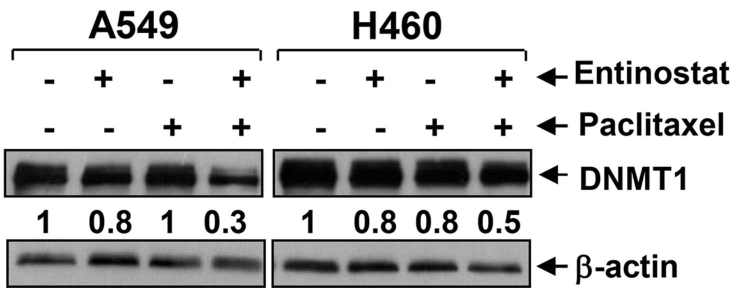

Supplementary Figure S5: Entinostat and/or paclitaxel reduce the expression levels of DNMT1 in NSCLC cells *in vitro* (related to Figure 9). A549 and H460 cells were treated with either entinostat (3 μmol/L for A549 and 0.5 μmol/L for H460), or paclitaxel (6 nmol/L and 3 nmol/L for A549 and H460, respectively) alone, or combinations of entinostat and paclitaxel for 24 h. Cells were collected and subjected to western blot analyses with specific antibodies directed against DNMT1 or β-actin. The densitometry analyses of DNMT1 signals were shown underneath, and the arbitrary numbers indicated the intensities of each cell line relative to controls, defined as 1.0.

Supplementary Table S1: Primers used for PCR analyses

Name	Sequence (5′ - 3′)	Amplicon (bp)	Annealing temp (°C)
**qRT-PCR**			
β-actin	F: AGAGCTA CGAGCTGCCTGAC	184	57
	R: AGCACTGTGTTGGCGTACAG		
Survivin	F: CCACCGCATCTCTACATTCA	184	57
	R: TATGTTCCTCTATGGGGTCG		
Bcl-xL	F: GTAAACTGGGGTCGCATTGT	198	57
	R: TGCTGCATTGTTCCCATAGA		
Mcl-1	F: GACGAGTTGTACCGGCAGTC	197	57
	R: ATGTCCAGTTTCCGAAGCAT		
**MSP**			
miR-203	F: GAGTATTTTCGGTTTAGACGAGAC	300	58
miR-203	R: CCTTTTATACGACGCAACCG		
**USP**			
miR-203	F: TTTGAGTATTTTTGGTTTAGATGAGAT	300	58
miR-203	R: AACACCTTTTATACAACACAACCA		

Supplementary Table S2: Clinical characteristics of 20 patients with NSCLC, related to Figure 11

**Age**		
	Range	42–72
	Mean	57.2
**Sex**	***n* (%)**	
	Female	6 (30)
	Male	14 (70)
**TNM stage**	***n* (%)**	
	T1	10 (50)
	T2	6 (30)
	T3	4 (20)

Supplementary Table S3: Clinical information of 20 patients with NSCLC, related to Figure 11

Patient	Age	Gender	TNM Stage	Tumor Histology Type
**1**	57	M	III	Large cell carcinoma
**2**	72	M	IIa	Adenocarcinoma
**3**	56	M	IIb	Adenocarcinoma
**4**	54	M	Ib	Squamous cell carcinoma
**5**	46	F	IIb	Adenocarcinoma
**6**	49	M	III	Squamous cell carcinoma
**7**	68	M	IIa	Squamous cell carcinoma
**8**	47	F	Ia	Adenocarcinoma
**9**	56	F	Ia	Squamous cell carcinoma
**10**	61	F	II	Adenocarcinoma
**11**	54	M	Ib	Squamous cell carcinoma

**12**	42	M	Ia	Squamous cell carcinoma
**13**	48	M	Ib	Adenocarcinoma
**14**	65	M	Ia	Adenocarcinoma
**15**	54	M	Ib	Squamous cell carcinoma
**16**	55	M	III	Squamous cell carcinoma
**17**	69	F	Ia	Adenocarcinoma
**18**	72	M	Ib	Squamous cell carcinoma
**19**	64	M	IIb	Adenocarcinoma
**20**	55	F	III	Squamous cell carcinoma

F: female; M: male; TNM: TNM-classification.

Supplementary Table S4: Clinical characteristics of 61 patients with NSCLC, related to Figure 10

**Age**		
	Range	23–76
	Mean	57.2
**Sex**	***n* (%)**	
	Female	30 (49.2)
	Male	31 (50.8)
**TNM stage**	***n* (%)**	
	T1	31 (50.8)
	T2	25 (41)
	T3	5 (8.2)
**IHC Positive Staining**	***n* (%)**	
	DNMT1 in NSCLC	29 (47.5)
	DNMT1 in normal adjacent lung tissue	22 (36)
	Survivin in NSCLC	29 (47.5)
	Survivin in normal adjacent lung tissue	23 (37.7)

Supplementary Table S5: Clinical information of 61 patients with NSCLC, related to Figure 10

Patient	Age	Gender	TNM Stage	Tumor Histology Type	DNMT1 IHC-T	DNMT1 IHC-N	Survivin IHC-T	Survivin IHC-N
**1**	71	M	III	Adenocarcinoma	2	0	2	0
**2**	45	F	IIa	Adenocarcinoma	0	0	0	0
**3**	47	F	Ia	Adenocarcinoma	1	1	1	1
**4**	33	F	II	Adenocarcinoma	0	0	0	0
**5**	38	F	Ia	Adenocarcinoma	1	0	1	0
**6**	55	M	III	Adenocarcinoma	2	1	3	1
**7**	76	M	Ia	Adenocarcinoma	0	0	0	0
**8**	58	M	IIb	Adenocarcinoma	3	0	3	0
**9**	43	F	Ib	Adenocarcinoma	0	0	0	0
**10**	61	M	IIa	Adenocarcinoma	0	0	0	0
**11**	65	M	IIa	Adenocarcinoma	0	0	0	0
**12**	46	F	Ia	Adenocarcinoma	0	0	0	0
**13**	59	M	Ia	Adenocarcinoma	2	1	1	1

**14**	60	F	IIb	Adenocarcinoma	2	0	2	0
**15**	61	M	Ib	Adenocarcinoma	0	0	0	0
**16**	49	M	Ia	Adenocarcinoma	1	0	1	0
**17**	75	M	IIa	Adenocarcinoma	2	2	1	2
**18**	68	F	II	Adenocarcinoma	1	1	1	1
**19**	65	M	Ia	Adenocarcinoma	0	1	0	1
**20**	54	F	IIb	Adenocarcinoma	0	0	0	0
**21**	66	M	Ib	Adenocarcinoma	2	1	2	1
**22**	51	F	II	Adenocarcinoma	1	2	2	2
**23**	54	F	II	Adenocarcinoma	0	0	0	0
**24**	67	F	I	Adenocarcinoma	0	0	0	0
**25**	66	M	I	Adenocarcinoma	0	0	0	0
**26**	72	F	II	Adenocarcinoma	0	0	0	0
**27**	72	M	IIa	Adenocarcinoma	2	1	1	1
**28**	69	M	II	Adenocarcinoma	0	0	0	0
**29**	42	F	IIb	Adenocarcinoma	2	1	2	1
**30**	71	F	Ia	Adenocarcinoma	1	0	1	0
**31**	52	M	Ib	Adenocarcinoma	0	1	0	1
**32**	52	M	III	Adenocarcinoma	3	0	3	0
**33**	62	F	Ib	Adenocarcinoma	1	0	1	1
**34**	75	M	IIa	Adenocarcinoma	3	2	1	2
**35**	60	M	Ib	Adenocarcinoma	2	1	2	1
**36**	23	F	IIb	Adenocarcinoma	0	0	0	0
**37**	66	F	Ib	Adenocarcinoma	0	0	0	0
**38**	74	M	Ia	Adenocarcinoma	1	0	1	0
**39**	75	M	IIa	Adenocarcinoma	0	2	0	2
**40**	59	F	Ia	Adenocarcinoma	0	0	0	0
**41**	46	F	Ib	Adenocarcinoma	1	0	1	0
**42**	53	M	Ia	Adenocarcinoma	2	1	2	1
**43**	41	F	Ia	Adenocarcinoma	0	0	0	0
**44**	53	M	IIa	Adenocarcinoma	3	1	2	1
**45**	49	F	Ib	Adenocarcinoma	0	0	0	0
**46**	39	M	III	Adenocarcinoma	0	0	0	0
**47**	74	M	III	Adenocarcinoma	0	0	0	0
**48**	47	F	Ia	Adenocarcinoma	0	0	0	0
**49**	60	F	IIb	Adenocarcinoma	0	0	0	0
**50**	55	F	III	Adenocarcinoma	0	0	0	0
**51**	55	F	Ia	Adenocarcinoma	1	1	1	1
**52**	46	M	IIa	Adenocarcinoma	2	2	2	2
**53**	43	M	I	Adenocarcinoma	0	0	0	0
**54**	56	M	IIa	Adenocarcinoma	2	2	2	2
**55**	45	F	IIa	Adenocarcinoma	0	0	0	0
**56**	68	M	IIa	Adenocarcinoma	1	1	1	1
**57**	72	M	IIa	Adenocarcinoma	1	2	1	2
**58**	60	F	Ib	Adenocarcinoma	0	1	0	1
**59**	49	F	Ib	Adenocarcinoma	0	0	0	0

**60**	52	F	Ia	Adenocarcinoma	3	0	3	0
**61**	72	M	IIa	Adenocarcinoma	0	0	0	0

F: female; M: male; TNM: TNM-classification; IHC-T: immunohistochemistry-tumor; IHC-N: immunohistochemistry-normal adjacent tissue. For DNMT1 or Survivin IHC, stained slides were scored according to the percentage of cells with positive staining for each antigen (0: 0~4%; 1: 5%~25%; 2: 26%~50%; and 3: ≥ 51%).

Supplementary Table S6: The spearman rank correlation between DNMT1 or Survivin and TNM stages, age, or gender of the patients with NSCLC, related to Figure 10

	**TNM stages**	**Age**	**Gender**
**Tumor DNMT1**	$r = 1$<br>$P < 0.0001$<br>($n = 61$)	$r = 1$<br>$P < 0.0001$<br>($n = 61$)	$r = 0.30175$<br>$P = 0.0181$<br>($n = 61$)
**Tumor Survivin**	$r = 1$<br>$P < 0.0001$<br>($n = 61$)	$r = 0.03068$<br>$P = 0.8144$<br>($n = 61$)	$r = 0.24337$<br>$P = 0.0588$<br>($n = 61$)
